# Plant viruses convergently target NPR1 with various strategies to suppress salicylic acid‐mediated antiviral immunity

**DOI:** 10.1111/jipb.13866

**Published:** 2025-02-21

**Authors:** Xue Jiang, Yingshuai Yang, Yong Li, Yongzhi Wang, Bernardo Rodamilans, Weiqin Ji, Xiaoxia Wu, Juan Antonio García, Xiaoyun Wu, Xiaofei Cheng

**Affiliations:** ^1^ College of Plant Protection, Northeast Agricultural University Harbin 150030 China; ^2^ College of Life Science, Northeast Agricultural University Harbin 150030 China; ^3^ Institute of Plant Protection, Jilin Academy of Agricultural Sciences Changchun 130033 China; ^4^ Departamento de Genética Molecular de Plantas, Centro Nacional de Biotecnología (CNB‐CSIC) Campus Universidad Autónoma de Madrid Madrid 28049 Spain

**Keywords:** alfalfa mosaic virus, beet severe curly‐top virus, degradation, NPR1, salicylic acid, virus

## Abstract

NONEXPRESSER OF PATHOGENESIS‐RELATED GENES 1 (NPR1), the receptor for salicylic acid (SA), plays a central role in the SA‐mediated basal antiviral responses. Recent studies have shown that two different plant RNA viruses encode proteins that suppress such antiviral responses by inhibiting its SUMOylation and inducing its degradation, respectively. However, it is unclear whether targeting NPR1 is a general phenomenon in viruses and whether viruses have novel strategies to inhibit NPR1. In the present study, we report that two different positive‐sense single‐stranded RNA (+ssRNA) viruses, namely, alfalfa mosaic virus (AMV) and potato virus X (PVX); one negative‐sense single‐stranded RNA (−ssRNA) virus (calla lily chlorotic spot virus, CCSV); and one single‐stranded DNA virus (beet severe curly‐top virus, BSCTV) that also encode one or more proteins that interact with NPR1. In addition, we found that the AMV‐encoded coat protein (CP) can induce NPR1 degradation by recruiting S‐phase kinase‐associated protein 1 (Skp1), a key component of the Skp1/cullin1/F‐box (SCF) E3 ligase. In contrast, the BSCTV‐encoded V2 protein inhibits NPR1 function, probably by affecting its nucleocytoplasmic distribution via the nuclear export factor ALY. Taken together, these data suggest that NPR1 is one of the central hubs in the molecular arms race between plants and viruses and that different viruses have independently evolved different strategies to target NPR1 and disrupt its function.

## Graphical abstract

Viral infection triggers monomerization and nuclear translocation of the salicylic acid receptor NONEXPRESSOR OF PATHOGENESIS‐RELATED GENES1 (NPR1) to activate pathogenesis‐related gene expression. Nevertheless, viruses have developed one or more proteins to target NPR1 and impair its functionality through mechanisms such as inducing its degradation, altering its nucleocytoplasmic distribution, or inhibiting its sumoylation.

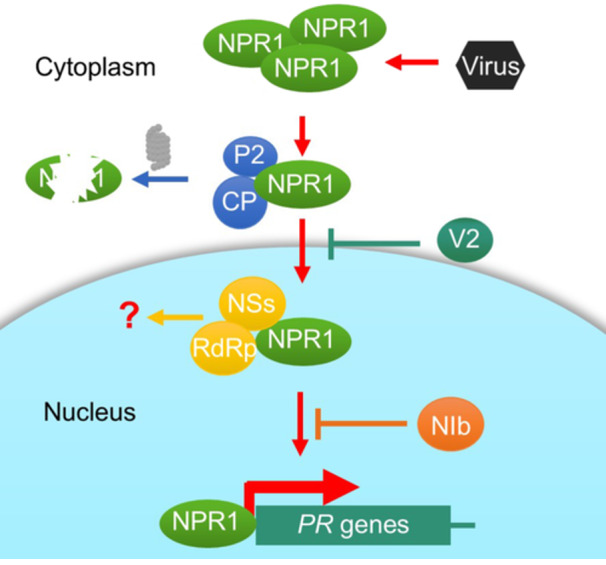

## INTRODUCTION

Salicylic acid (SA) is one of the most important defense‐related phytohormones in plants (Vlot et al., 2009). The invasion of phytopathogens, such as bacteria, viruses, oomycetes, or fungi, induces SA accumulation in both local and distal non‐infected tissues, leading to the expression of a group of pathogenesis‐related (PR) genes. Blocking SA accumulation, either by knocking out the genes involved in SA synthesis, accumulation, or signaling, results in compromised resistance (Huang et al., 2005; Mayers et al., 2005; Baebler et al., 2011; Villanueva‐Alonzo et al., 2013; Zhu et al., 2014), whereas the exogenous application of SA or its analogs increases resistance to various pathogens (Murphy et al., 1999; Singh et al., 2004). In plants, SA is perceived by a class of BTB/POZ (Broad‐complex, Tramtrack, and Bric‐a‐brac/Pox virus and Zinc finger) and ankyrin repeat domain‐containing proteins, namely NONEXPRESSER OF PATHOGENESIS‐RELATED GENES 1‒6 (NPR1‒6) (Castelló et al., 2018). NPR1 and possibly NPR2 positively regulate SA (Cao et al., 1994; Castelló et al., 2018), whereas NPR3 and NPR4 negatively regulate defense (Zhang et al., 2006; Fu et al., 2012; Ding et al., 2018). NPR1 is usually present as large oligomers in the cytoplasm under steady‐state conditions (Kumar et al., 2022). Activation of plant immunity during pathogen invasion rapidly alters the cellular redox state and induces the accumulation of SA via *de novo* biosynthesis and hydrolysis of inactivated forms (Ding and Ding, 2020), resulting in the monomerization of cytoplasmic NPR1 (Tada et al., 2008). NPR1 monomers then enter nuclei (Lee et al., 2015), where they are modified sequentially by various post‐translational modifications, including SUMOylation, phosphorylation, and ubiquitination, and then activate the expression of PR genes via the transcription factors TGACG SEQUENCE‐SPECIFIC BINDING PROTEIN (TGA) TGA2/TGA5/TGA6 (Zhang et al., 1999; Despres et al., 2000; Fan and Dong, 2002; Spoel et al., 2009; Skelly et al., 2019; Nomoto et al., 2021).

The function of SA signaling is thought to be similar in monocots as the knockout of NPR1 homologs in rice and barley also attenuates resistance to several pathogens (Chern et al., 2001; Li et al., 2020). However, the SA signaling pathway differs slightly between monocots and dicots, e.g., SA levels are much higher in monocots than in dicots under steady‐state conditions, and the SA and JA pathways are generally antagonistic in dicots but synergistic in monocots through the interplay between NPR1 and JASMONATE ZIM‐DOMAIN (JAZ) proteins (Nomoto et al., 2021; Zhang et al., 2023). Nevertheless, NPR1 is also thought to confer broad‐spectrum antiviral function in monocots (Chern et al., 2001; Liu et al., 2017; Li et al., 2020).

Given the important role of NPR1 in PR gene expression, it is not surprising that some pathogens may have evolved effectors that target NPR1. For instance, RxLR48 of *Phytophthora capsica*, AvrPtoB of *Pseudomonas syringae*, and PUCCINIA NPR1 INTERACTOR (PNPi) of *Puccinia striiformis* interact with NPR1 and inhibit its function by destabilizing it, sequestering it in the nucleus, or disrupting the NPR1–TGA1 interaction (Wang et al., 2016; Chen et al., 2017; Li et al., 2019). Recently, we found that the RNA‐dependent RNA polymerase (NIb) of turnip mosaic virus, a positive‐sense single‐stranded RNA (+ssRNA) virus of the family *Potyviridae* in the order *Picornavirales*, interacts with NPR1 and suppresses its activity by inhibiting its SUMOylation (Liu et al., 2023). In addition, another study found that the p2 protein of rice stripe virus (RSV), a negative‐sense single‐stranded RNA (−ssRNA) virus of the family *Phenuiviridae* in the order *Bunyavirales*, promotes OsNPR1 degradation by enhancing its association with OsCUL3a (Zhang et al., 2023). However, whether other viruses have also evolved proteins that target NPR1 and whether there are additional mechanisms to suppress NPR1 function remain elusive. In this study, we further report that several unrelated plant +ssRNA, −ssRNA, and DNA viruses also encode proteins that target NPR1 and two novel strategies to suppress NPR1‐mediated antiviral responses.

## RESULTS

### SA signaling pathway has a broad‐spectrum antiviral role

**Figure 1:**
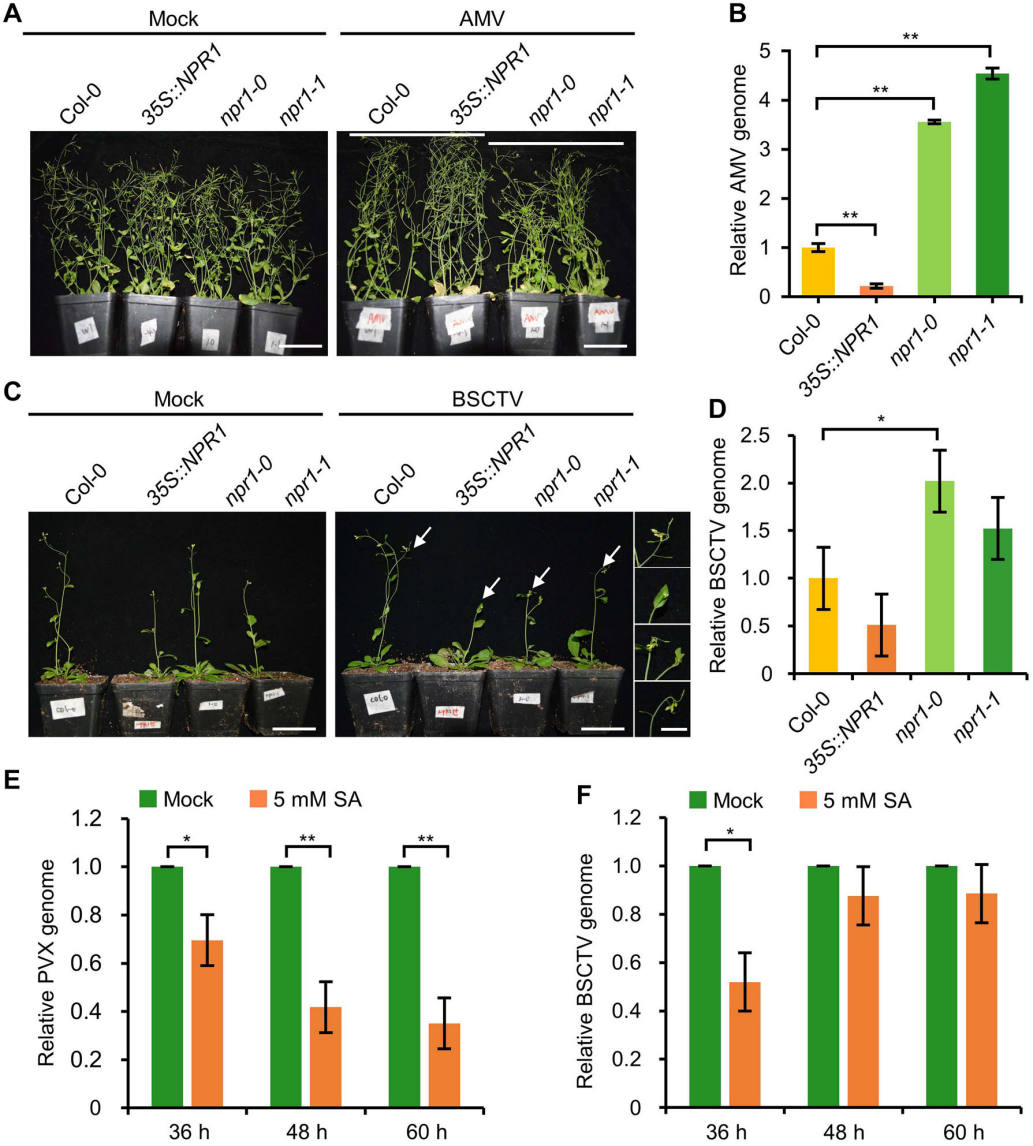
SA signaling has a broad‐spectrum antiviral role **(A)** Phenotypes of wild‐type (Col‐0), *35S::NPR1‐GFP* (*35S::NPR1*), *npr1‐0*, and *npr1‐1* seedlings infected with AMV at 4 wpi. Scale bars = 5 cm. **(B)** Bar graph showing the accumulation of AMV genomic RNA in Col‐0, *35S::NPR1*, *npr1‐0*, and *npr1‐1* seedlings infected with AMV at 4 wpi (*n* = 3), determined by RT‐qPCR. One asterisk (*) indicates a *P*‐value less than 0.05 (*P* < 0.05), while two asterisks (**) indicates a *P*‐value less than 0.01 (*P* < 0.01). The same standard is applied to the following figures. **(C)** Phenotypes of Col‐0, *35S::NPR1*, *npr1‐0*, and *npr1‐1* seedlings infected with BSCTV at 12 dpi. The white arrows indicate the enlarged curled pedicel in the right penal (from top to bottom are Col‐0, *35S::NPR1*, *npr1‐0*, and *npr1‐1*, respectively). Scale bars = 5 cm (main panels) or 1 cm (enlarged panels). **(D)** Bar graph showing the accumulation of BSCTV DNA in BSCTV‐infected Col‐0, *35S::NPR1*, *npr1‐0*, and *npr1‐1* seedlings at 12 dpi (*n* = 3), determined by qPCR. **(E**, **F)** Bar graphs showing the accumulation of PVX genomic RNA **(E)** and BSCTV genomic DNA **(F)**, determined by RT‐qPCR and qPCR, respectively, in *N. benthamiana* leaves treated with water or 5 mM SA at the indicated time points (*n* = 3).

Previously, we found that NPR1 plays a critical role in restricting the compatible infection by TuMV (Liu et al., 2023). To further investigate the function of SA signaling in the compatible plant–virus interactions, we analyzed the infectivity of two different plant viruses, namely alfalfa mosaic virus (AMV) and beet severe curly‐top virus (BSCTV), on wild‐type *Arabidopsis thaliana* ecotype Columbia 0 (Col‐0; WT), two *npr1* knockout mutants (*npr1‐0* and *npr1‐1*), and a transgenic line overexpressing a C‐terminal GFP‐tagged NPR1 (*35S::NPR1‐GFP*) (Liu et al., 2023). AMV is a +ssRNA virus of the genus *Alfamovirus* (family *Bromoviridae*, order *Martellivirales*), whereas BSCTV is a circular single‐stranded DNA virus of the genus *Curtovirus* (family *Geminiviridae*, order *Geplafuvirales*). Three‐week‐old seedlings were inoculated with AMV and an infectious cDNA clone of BSCTV by mechanical inoculation and agroinfiltration, respectively. At 4 weeks post‐inoculation (wpi), we found that WT and *35S::NPR1‐GFP* seedlings inoculated with AMV looked similar to mock seedlings (Figure 1A), further confirming that Arabidopsis is a symptomless host of AMV (Balasubramaniam et al., 2006). Interestingly, virus‐infected seedlings of *npr1‐0* and *npr1‐1* were slightly smaller than mock‐treated seedlings of the same genotype (Figure 1A). Reverse transcription followed by quantitative polymerase chain reaction (RT‒qPCR) showed that the amount of genomic RNA of AMV was significantly decreased in *35S::NPR1‐GFP* and increased in *npr1‐0* and *npr1‐1* seedlings (Figure 1B). We also found that the stem and pedicel of BSCTV‐infected *npr1‐0* and *npr1‐1* seedlings were severely curled compared with those of BSCTV‐infected WT and *35S::NPR1‐GFP* seedlings at 12 d post‐inoculation (dpi) (Figure 1C). Compared with WT, the seedlings of *npr1‐0* and, to a lesser extent, *npr1‐1* accumulated higher levels of BSCTV DNA, whereas *35S::NPR1‐GFP* contained lower levels of BSCTV DNA as determined by qPCR (Figure 1D). These data suggest that the NPR1‐mediated SA signaling pathway also plays a role in limiting AMV and BSCTV infection.

We further tested the antiviral function of the SA signaling pathway by spraying SA directly on another host plant (*Nicotiana benthamiana*). BSCTV and potato virus X (PVX), a +ssRNA virus of the genus *Potexvirus* (family *Alphaflexiviridae*, order *Tymovirales*), were used as the model viruses. Leaves of 4‐week‐old *N. benthamiana* seedlings were agroinfiltrated with PVX or BSCTV infectious clone and then treated with water (mock) or 5 mM of SA. The accumulation of viral RNA (for PVX) or DNA (for BSCTV) in the inoculated leaves was determined by RT‐qPCR or qPCR at 36, 48, and 60 h post‐inoculation (hpi), respectively. The results showed that the accumulation of the PVX RNA and BSCTV DNA in SA‐treated leaves was significantly lower than that in the mock‐treated samples at 36 hpi (Figure 1E, F), indicating that the SA signaling pathway also restricts PVX and BSCTV infection in *N. benthamiana* at the early infection stage. The levels of PVX RNA were consistently lower in SA‐treated leaves than in those treated with inoculation buffer alone at 48 and 60 hpi; however, the amount of BSCTV DNA was increased to a level similar to that of the mock‐treated leaves at 48 and 60 hpi (Figure 1E, F), suggesting that BSCTV may be able to almost completely suppress the SA‐mediated resistance or that the infection process of BSCTV is less affected by SA‐mediated resistance. Taken together, these data suggest that the SA signaling pathway may play a broad‐spectrum antiviral role in dicotyledonous plants.

### AMV CP interacts with the N‐terminal domain of NPR1

**Figure 2:**
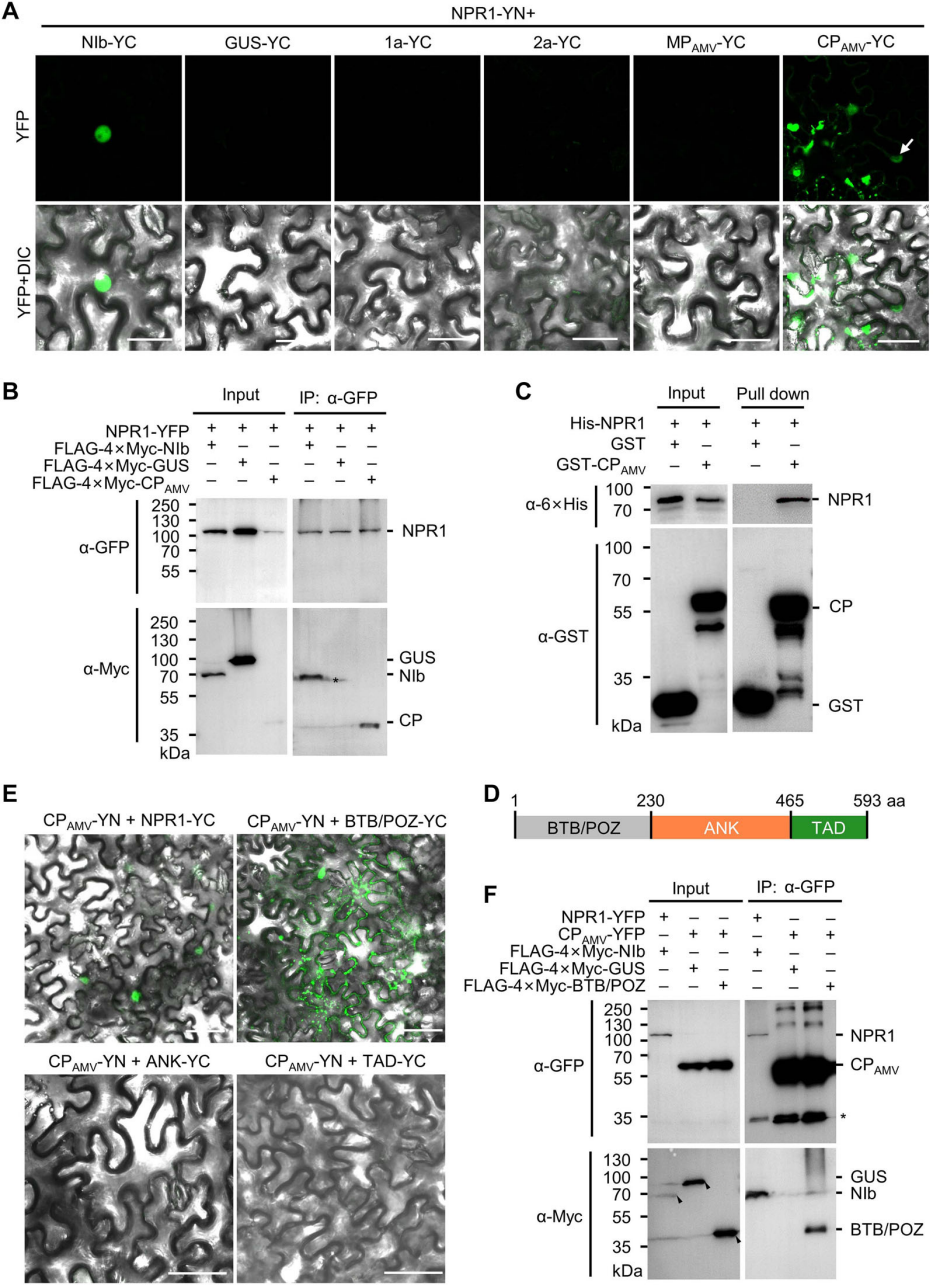
AMV CP interacts with NPR1 **(A)** BiFC assay showing the interactions between NPR1‐YN and YC‐tagged AMV‐encoded 1a, 2a, MP (MP_AMV_), and CP (CP_AMV_) in *N. benthamiana* epidermal cells. Proteins were expressed by agroinfiltration, and photographs were taken at 2 dpi with identical settings; the white arrow indicates the nucleus; DIC, differential interference contrast channel; scale bars = 50 μm. **(B)** Co‐IP assay for the protein–protein interaction between NPR1‐YFP and FLAG‐4×Myc‐tagged CP_AMV_, GUS, or TuMV NIb. Proteins were expressed by agroinfiltration in *N. benthamiana* leaves and affinity purified by GFP‐Trap agarose at 42 hpi. NPR1‐YFP and FLAG‐4×Myc‐tagged proteins were detected using polyclonal antibodies against the N‐terminal GFP (*α*‐GFP) and Myc tag (*α*‐Myc), respectively. The nonspecific band is indicated by an asterisk. **(C)** Pull‐down assay by retention in glutathione agarose for the interaction between 6×His‐NPR1 and GST or GST‐CP_AMV_. GST, GST‐CP_AMV_, and 6×His‐NPR1 were detected using monoclonal antibodies against GST (*α*‐GST) and 6×His‐tag (*α*‐6×His), respectively. Asterisks indicate nonspecific bands. **(D)** Representation of the NPR1 domain arrangement. Numbers represent the amino acid positions of domain boundaries. **(E)** BiFC assay for the interactions between CP_AMV_‐YN and YC‐tagged truncated NPR1 mutants in *N. benthamiana* epidermal cells at 2 dpi. Scale bars = 50 μm. **(F)** Co‐IP for the protein–protein interaction between CP_AMV_‐YFP and the FLAG‐4×Myc‐tagged GUS and the BTB/POZ domains of NPR1. Proteins were expressed via agroinfiltration in *N. benthamiana* leaves and affinity purified by GFP‐Trap agarose at 2 dpi. Asterisks indicate nonspecific bands.

Given the important antiviral role of SA‐mediated immunity, and the fact that some pathogens, e.g., *Pseudomonas syringae*, *Phytophthora capsica*, *Puccinia striiformis*, RSV, and TuMV, have evolved effectors to target NPR1 (Wang et al., 2016; Chen et al., 2017; Li et al., 2019; Liu et al., 2023; Zhang et al., 2023), we hypothesized that most plant viruses have evolved one or more viral proteins to interact with NPR1 and disrupt its function. Therefore, we investigated the interactions between AMV‐encoded proteins and NPR1 in a bimolecular fluorescence complementation (BiFC) assay. The tripartite genome of AMV encodes four proteins, namely 1a, 2a, coat protein (CP_AMV_), and movement protein (MP_AMV_). We transiently expressed NPR1 tagged with the N‐terminal portion of YFP (NPR1‐YN) and the AMV‐encoded proteins tagged with the C‐terminal portion of YFP (YC) under the control of the cauliflower mosaic virus (CaMV) 35S promoter in *N. benthamiana* leaves by agroinfiltration. TuMV‐encoded NIb was included as a positive control, while *β*‐glucuronidase (GUS) was used as a negative control. Consistent with our previous study (Liu et al., 2023), the nuclei of *N. benthamiana* epidermal cells expressing NPR1‐YN and NIb‐YC showed a bright YFP signal in the nucleus, while the cells expressing NPR1‐YN and GUS‐YC showed no fluorescence under the same conditions (Figure 2A). Interestingly, a bright YFP signal was found in the cells coexpressing NPR1‐YN and CP_AMV_‐YC, but not 1a‐YC, 2a‐YC, or MP_AMV_‐YC (Figure 2A). Noticeably, the YFP signal from the interaction between NPR1‐YN and CP_AMV_‐YC was found not only in the nuclei but also in the cytoplasm as condensates (Figure 2A). Western blotting confirmed that the absence of fluorescence for the other viral proteins was not due to their non‐expression (Figure S1). To further confirm the interaction between NPR1 and AMV CP, coimmunoprecipitation (Co‐IP) was performed. YFP‐tagged NPR1 (NPR1‐YFP) was transiently coexpressed with FLAG‐4×Myc‐tagged TuMV NIb (FLAG‐4×Myc‐NIb), AMV CP (FLAG‐4×Myc‐CP_AMV_), or GUS (FLAG‐4×Myc‐GUS) under the CaMV 35S promoter in *N. benthamiana* leaves. At 42 hpi, NPR1‐YFP was affinity purified with GFP‐Trap agarose and then analyzed by western blotting using polyclonal antibodies against the N‐terminal domain of GFP and Myc. The results showed that FLAG‐4×Myc‐NIb and FLAG‐4×Myc‐CP_AMV_, but not FLAG‐ 4×Myc‐GUS, were coimmunoprecipitated with NPR1‐YFP (Figure 2B). We further tested whether AMV CP physically interacted with NPR1 using purified 6×Histidine‐tagged NPR1 (6×His‐NPR1), glutathione *S*‐transferase (GST), and GST‐tagged CP_AMV_ (GST‐CP_AMV_) from *Escherichia coli*. The result showed that 6×His‐NPR1 was successfully pulled down by GST‐CP_AMV_ but not by the GST tag (Figure 2C). Taken together, these data indicated that AMV CP physically interacts with NPR1.

We also attempted to determine the domain of NPR1 that interacts with AMV CP. Based on its structure, NPR1 was divided into three non‐overlapping domains, namely the N‐terminal BTB/POZ domain, the central ankyrin repeat (ANK) domain, and the C‐terminal transcriptional activation domain (TAD) (Figure 2D). The interactions between AMV CP and the three domains of NPR1 were analyzed using BiFC. A bright YFP signal was found in *N. benthamiana* epidermal cells coexpressing CP_AMV_‐YN and BTB/POZ‐YC, but not in the cells coexpressing CP_AMV_‐YN plus ANK‐YC or TAD‐YC (Figures 2E, S2). A Co‐IP assay with transiently expressed proteins further confirmed the interaction between AMV CP and the BTB/POZ domain of NPR1 (Figures 2F, S3). Taken together, these data indicated that AMV CP targets the N‐terminal BTB/POZ domain of NPR1.

### AMV CP induces the degradation of NPR1

**Figure 3:**
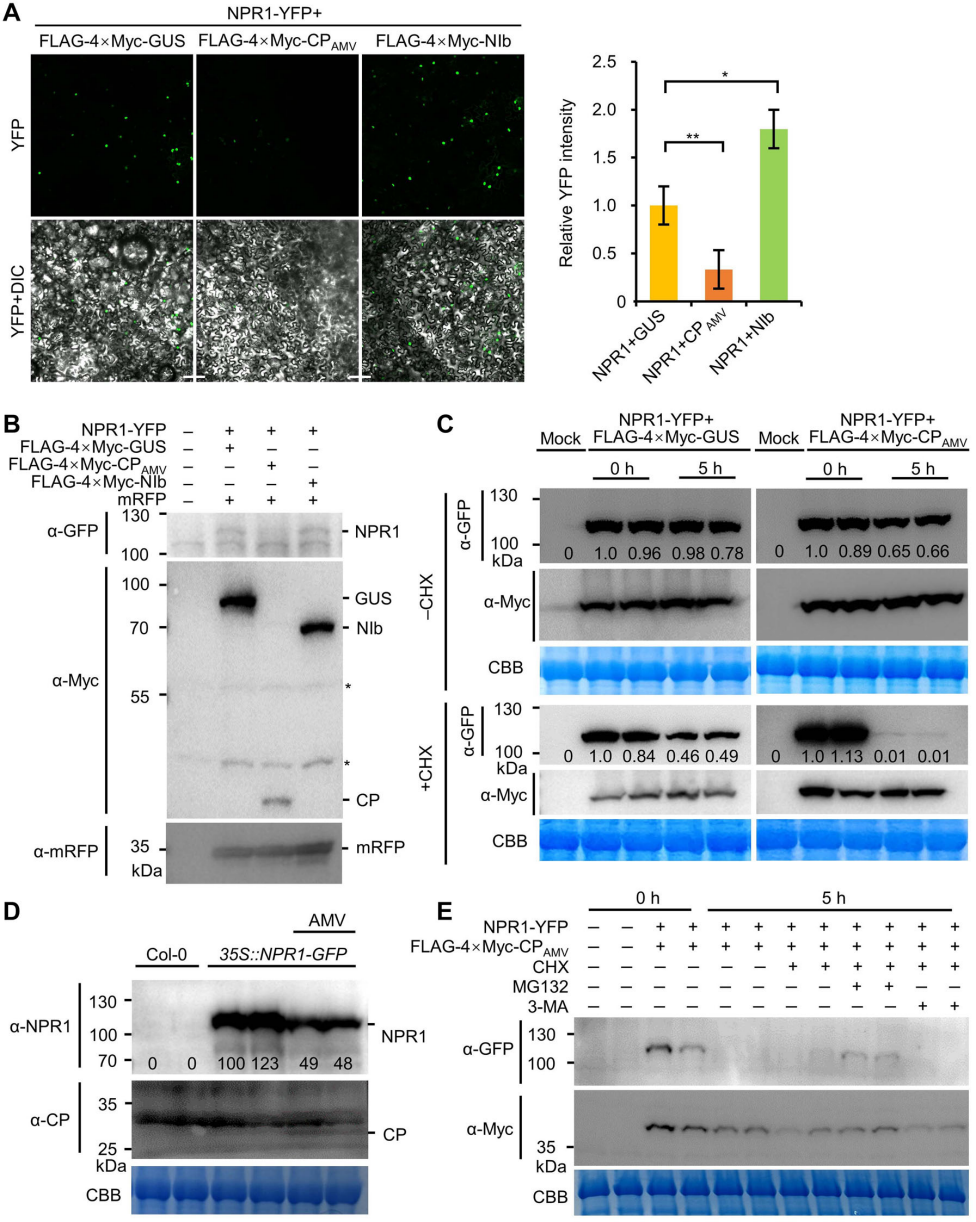
AMV CP destabilizes NPR1 **(A)** Confocal microscopy images comparing the fluorescence intensity of NPR1‐YFP coexpressed with FLAG‐4×Myc‐NIb, FLAG‐4×Myc‐CP_AMV_, or FLAG‐4×Myc‐GUS in *N. benthamiana* epidermal cells at 48 hpi. The bar graph in the right panel shows the average nuclear YFP signal intensity of three photographs. Equal amounts of agrobacteria harboring NPR1‐YFP were used in all combinations, and all photographs were taken with identical settings. Scale bars = 50 μm * and ** indicate *P* < 0.05 and *P* < 0.01 in Student's *t*‐test, respectively. **(B)** Western blotting showing the accumulation of NPR1‐YFP in *N. benthamiana* epidermal cells coexpressing FLAG‐4×Myc‐NIb, FLAG‐4×Myc‐CP_AMV_, or FLAG‐4×Myc‐GUS at 48 hpi. Asterisks indicate nonspecific bands. **(C)** Western blotting showing the level of NPR1‐YFP coexpressed with FLAG‐4×Myc‐CP_AMV_ or FLAG‐4×Myc‐GUS before or after cycloheximide (CHX) treatment for 5 h. The number under each lane represents the relative intensity of the band to the control. **(D)** Western blotting to assess the accumulation of NPR1‐YFP in leaves of mock or AMV‐infected *35S::NPR1‐GFP* seedlings at 21 dpi. NPR1‐YFP and AMV CP were detected using polyclonal anti‐NPR1 and monoclonal anti‐CP_AMV_, respectively. The number under each lane represents the relative intensity of the band to the control. **(E)** Western blotting to assess the accumulation of NPR1‐YFP and FLAG‐4×Myc‐CP_AMV_ coexpressed in *N. benthamiana* cells before or after treatment with MG132 or 3‐MA for 5 h.

We noticed that the amount of NPR1 protein was always lower when coexpressed with AMV CP than when coexpressed with TuMV NIb or GUS (Figure 2B). Therefore, we hypothesized that AMV CP might cause NPR1 degradation. To confirm this hypothesis, we directly compared the fluorescence intensity of transiently expressing NPR1‐YFP and FLAG‐4×Myc‐CP_AMV_, FLAG‐4×Myc‐NIb, or FLAG‐4×Myc‐GUS in the epidermal cells of *N. benthamiana*. The results showed that, in cells coexpressing FLAG‐4×Myc‐CP_AMV_, the fluorescence of NPR1‐YFP in the nucleus decreased to about one‐third of that in cells coexpressing FLAG‐4×Myc‐GUS, while NPR1 fluorescence in the nucleus was increased in cells expressing NIb (Figure 3A). Western blotting confirmed that the level of NPR1‐YFP was significantly decreased in the presence of FLAG‐4×Myc‐CP_AMV_ compared with FLAG‐4×Myc‐NIb or FLAG‐4×Myc‐GUS (Figure S4). To exclude the effects of different leaves on AMV CP accumulation, we repeated the above experiment together with a vector expressing free mRFP to show the transient expression efficiency. The results showed that the level of NPR1‐YFP was also significantly reduced in the presence of FLAG‐4×Myc‐CP_AMV_ compared with FLAG‐4×Myc‐NIb or FLAG‐4×Myc‐GUS (Figure 3B), indicating that AMV CP induces the degradation of NPR1. To further confirm this possibility, we transiently coexpressed NPR1‐YFP with FLAG‐4×Myc‐GUS or FLAG‐4×Myc‐CP_AMV_ in *N. benthamiana* leaves, treated the leaves with cycloheximide (CHX), the inhibitor of protein translation, or buffer at 43 hpi to inhibit protein translation, and then analyzed the degradation of NPR1 after 5 h of treatment. The results showed that, when coexpressing with GUS, the NPR1 level was almost unchanged at 0 h and 5 h, whereas when coexpressing with CP, the level of NPR1 decreased by 35% at 5 h compared with 0 h. However, after 5 h of CHX treatment, the amount of NPR1‐YFP was about half that before treatment when FLAG‐4×Myc‐GUS was coexpressed, but NPR1 was almost undetectable in the presence of FLAG‐4×Myc‐CP_AMV_ (Figure 3C), confirming that AMV CP induces NPR1 degradation. To confirm this possibility *in planta*, we infected *35S::NPR1‐GFP* seedlings with AMV and then compared the level of NPR1 in the control and AMV‐infected plants by western blotting at 21 dpi. The results showed that NPR1 levels after AMV infection decreased by about 50% compared with control plants (Figure 3D). Together, we concluded that AMV CP is capable of destabilizing NPR1.

Ubiquitin‐26S proteasome and autophagy are the two major protein degradation pathways in plants (Raffeiner et al., 2023). To explore the possible pathway that is involved in the AMV CP‐mediated degradation of NPR1, carbobenzoxy‐L‐leucyl‐L‐leucyl‐L‐leucinal (MG132) and 3‐methyladenine (3‐MA), two well known inhibitors of the ubiquitin‐26S proteasome and autophagy, respectively, were used (Cheng and Wang, 2017). We tested the effect of MG132 and 3‐MA on the accumulation of NPR1 and AMV CP in the presence of CHX in *N. benthamiana* leaves coexpressing NPR1‐YFP and FLAG‐4×Myc‐CP_AMV_. The results showed that the protein levels of NPR1 were increased only after MG132 treatment but not 3‐MA treatment (Figure 3E), indicating that the AMV CP‐mediated degradation of NPR1 is dependent on the ubiquitin‐26S proteasome pathway.

### AMV CP recruits Skp1 to degrade NPR1

**Figure 4:**
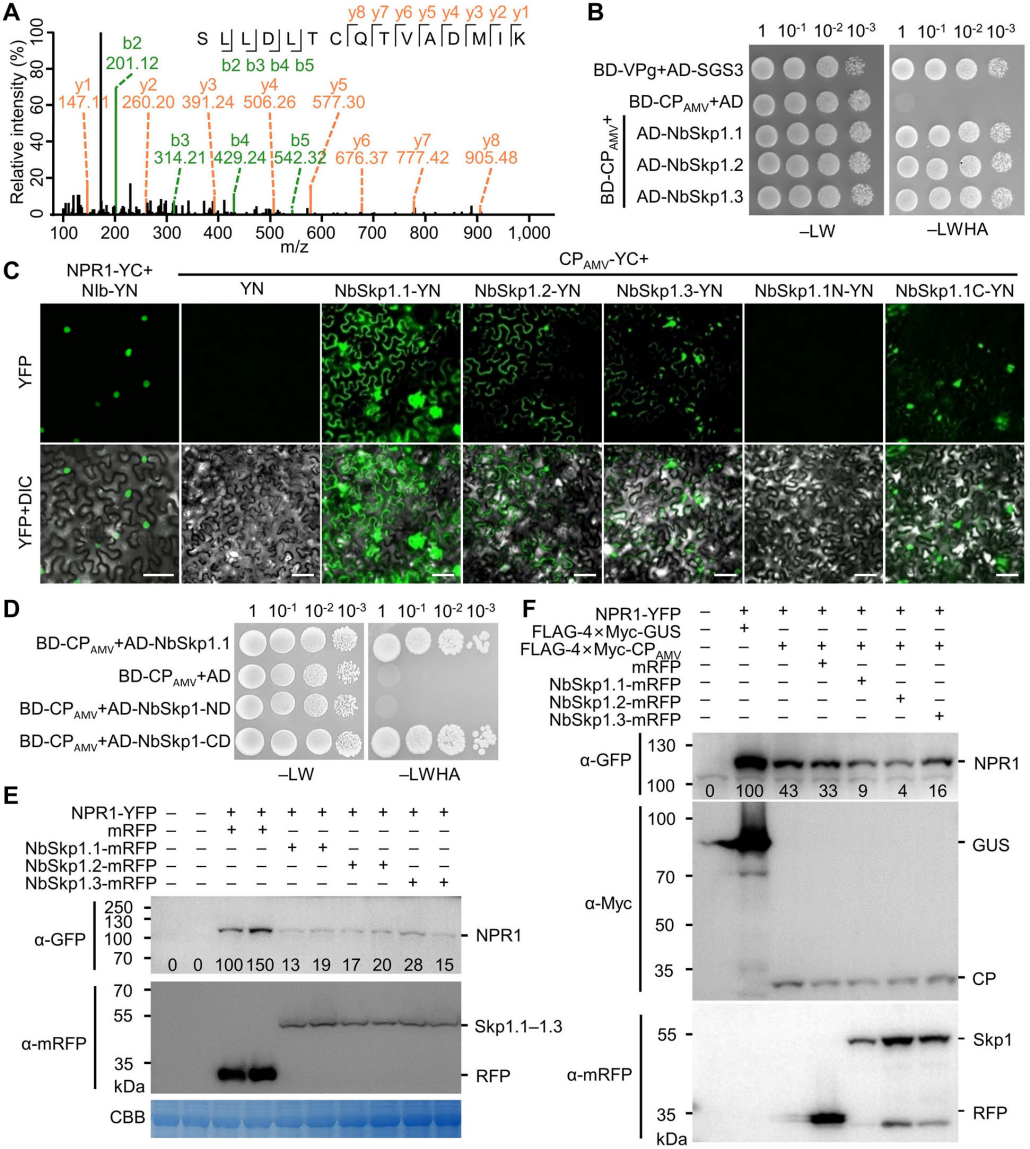
AMV CP induces the degradation of NPR1 via Skp1 **(A)** MS/MS spectrum of a NbSkp1 peptide detected in *N. benthamiana* leaves transiently expressing FLAG‐4×Myc‐CP_AMV_ and NPR1‐YFP. The x and y axes represent the mass‐to‐charge ratio (m/z) and relative intensity (%), respectively. The peptide sequence is shown on top with the collision‐induced fragmentation pattern. The b and y ions are shown in green and orange, respectively, while black peaks correspond to unexpected fragmentation events or noise. **(B)** Growth of serially diluted yeast cells expressing the DNA‐binding domain of GAL4 (BD) or BD‐tagged TuMV VPg or AMV CP plus the activation domain of GAL4 (AD) or AD‐tagged SGS3, NbSkp1.1, NbSkp1.2, or NbSkp1.3 on SD medium lacking leucine and tryptophan (−LW) or leucine, tryptophan, histidine, and adenine (−LWHA). **(C)** Confocal microscopy images of *N. benthamiana* epidermal cells expressing the indicated recombinant proteins at 2 dpi. Scale bars = 50 μm. **(D)** Growth of serially diluted yeast cells expressing the indicated recombinant proteins on −LW or −LWHA medium. **(E)** Western blotting to assess protein accumulation in *N. benthamiana* cells coexpressing NPR1‐YFP with mRFP or mRFP‐tagged NbSpk1.1, NbSkp1.2, or NbSkp1.3. NPR1‐YFP was detected with anti‐GFP and mRFP‐tagged recombinant proteins were detected using monoclonal anti‐mRFP antibody (*α*‐mRFP). The number under each lane represents the relative intensity of the band to the control. **(F)** Western blotting analysis of the accumulation of NPR1‐YFP in the *N. benthamiana* cells coexpressing FLAG‐4×Myc‐CP_AMV_ and mRFP‐tagged NbSkp1.1, NbSkp1.2, or NbSkp1.3. The number under each lane represents the relative intensity of the band compared with the control.

To explore the molecular mechanism of AMV CP‐induced NPR1 degradation, we transiently coexpressed FLAG‐4×Myc‐CP_AMV_ and NPR1‐YFP in *N. benthamiana* leaves. At 2 dpi, FLAG‐4×Myc‐CP_AMV_ was affinity purified and analyzed by tandem mass spectrometry (MS/MS). An affinity‐purified FLAG‐4×Myc tag that was coexpressed with NPR1‐YFP was used as a negative control. In total, 1,234 peptides belonging to 445 proteins were identified, including AMV CP and NPR1‐YFP (Table S1), further confirming the interaction between AMV CP and NPR1. Interestingly, a group of proteins belonging to the ubiquitin‐26S proteasome pathway was identified, namely ubiquitin, *S*‐phase kinase‐associated protein 1 (Skp1) of the SKP1/cullin1/F‐box (SCF) E3 ligase (Figure 4A), and four components of the 26S proteasome (PSMB6, PSMA4, RPN8, and PAF1), whereas no autophagy‐related protein was identified (Tables 1, S1), further suggesting that AMV CP induces NPR1 degradation via the ubiquitin‐26S proteasome pathway and that AMV CP may recruit a SCF E3 ligase to induce NPR1 degradation. There are three homologs of the human Skp1 (*NbSkp1.1*, *NbSkp1.2*, and *NbSkp1.3*) in the *N. benthamiana* genome (Jia et al., 2016). Therefore, we cloned all of them and tested their ability to interact with AMV CP by Y2H. The results showed that the yeast cells expressing the DNA‐binding domain of GAL4 (BD)‐tagged AMV CP (BD‐CP_AMV_) and the activation domain (AD)‐tagged NbSkp1.1, NbSkp1.2, or NbSkp1.3 survived on the medium lacking leucine, tryptophan, histidine, and adenine, while those expressing BD‐CP_AMV_ and AD failed to proliferate (Figure 4B). BiFC was further performed to confirm the interactions between AMV CP and *N. benthamiana* Skp1 homologs. The results showed that the epidermal cells of *N. benthamiana* leaves expressing CP_AMV_‐YC and YN‐tagged NbSkp1.1, NbSkp1.2, or NbSkp1.3 exhibited a bright YFP signal, whereas no YFP signal was found in the cells expressing CP_AMV_‐YC and YN (Figure 4C). Notably, cells expressing CP_AMV_‐YC and NbSkp1.1‐YN had the brightest fluorescence intensity, suggesting that AMV CP and NbSkp1.1 had the strongest interactions. We also cloned the two Arabidopsis Skp1 homologs (*AtASK1* and *AtASK2*) (Risseeuw et al., 2003) and analyzed their interaction with AMV CP using BiFC. The results showed that a bright fluorescence signal was recorded in the epidermal cells expressing CP_AMV_‐YC and AtASK1‐YN or AtASK2 (Figure S5), indicating that AMV CP also interacts with Arabidopsis Skp1 homologs.

**Table 1:** Proteins of the UPS pathway that were identified by MS/MS

Gene name	Unique peptide	Coverage (%)	Score
SKP1‐like protein 1 (Skp1.1)	2	14.1	17.807
Ubiquitin (Ubi)	2	83.6	128.92
26S proteasome subunit beta type‐6 (PSMB6)	7	51.4	215.48
26S proteasome subunit alpha 4 (PSMA4)	8	53.7	187.21
26S proteasome regulatory subunit RPN8 (RPN8)	1	12.7	6.94
26S proteasome subunit alpha type 1 (PAF1)	11	39.4	323.31

The N‐terminal portion of Skp1 is involved in cullin‐1 (CUL1) binding, while the C‐terminal portion is required for interaction with F‐box proteins that respond to substrate recognition (Ban and Estelle, 2021). BiFC and Y2H assays revealed that AMV CP interacts with the C‐terminal domain of NbSkp1.1 (Figure 4C, D). A previous study showed that a HOS15‐containing SKP1/Cullin1 E3 ligase interacts with and destabilizes NPR1 under unchallenged conditions (Shen et al., 2020a). We therefore tested whether Skp1 alone could induce the degradation of NPR1 using a transient protein degradation assay. We found that the protein level of NPR1‐YFP was apparently reduced in the presence of mRFP‐tagged NbSkp1.1, NbSkp1.2, and NbSkp1.3 compared with mRFP (Figure 4E). Moreover, the coexpression of NbSkp1.1, NbSkp1.2, or NbSkp1.3 could further accelerate the degradation of NPR1 by AMV CP (Figure 4F). We further tested whether AtASK1 or AtASK2 could induce the degradation of NPR1. A transient protein degradation assay showed that NPR1‐YFP was dramatically reduced when coexpressed with AtASK1‐mRFP or AtASK2‐mRFP compared with mRFP (Figure S6A). Furthermore, the coexpression of AtASK1 or AtASK2 could further accelerate the degradation of NPR1 by AMV CP (Figure S6B). Taken together, these data indicated that AMV CP induces the degradation of NPR1 through the recruitment of Skp1.

### BSCTV V2 interacts with NPR1

**Figure 5:**
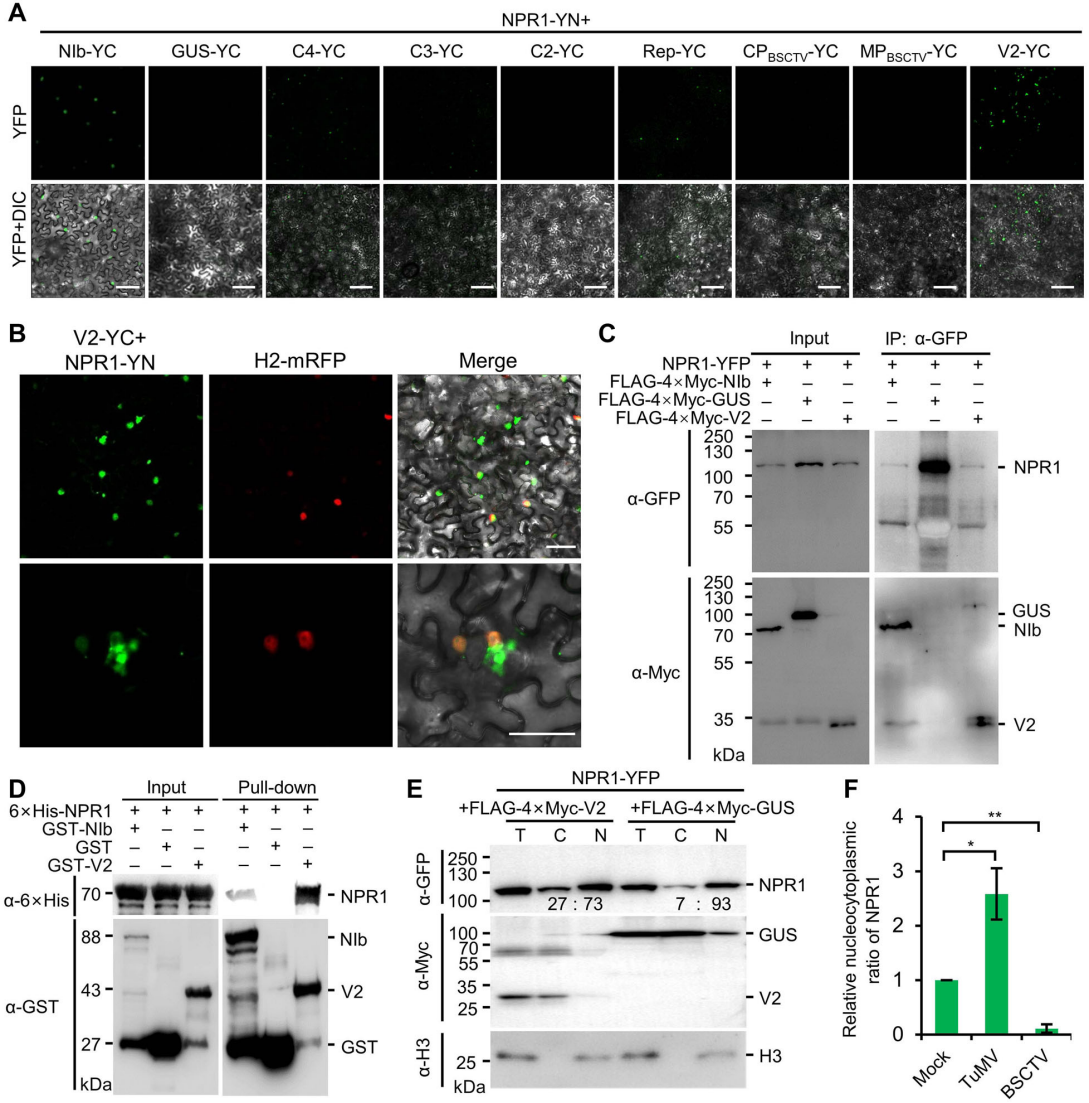
BSCTV V2 interacts with NPR1 **(A)** Confocal microscopy images of *N. benthamiana* epidermal cells coexpressing NPR1‐YN and YC‐tagged BSCTV‐encoded proteins at 2 dpi. Scale bars = 50 μm. **(B)** Localization of the YFP signal from the interaction between V2‐YC and NPR1‐YN in *N. benthamiana* epidermal cells at 2 dpi. Nuclei are indicated by mRFP‐tagged histone H2 (H2‐mRFP). Scale bars = 50 μm. **(C)** Co‐IP results for the interaction between NPR1‐YFP and FLAG‐4×Myc‐tagged TuMV NIb, GUS, and BSCTV V2. **(D)** Pull‐down assay by retention in glutathione agarose for the interaction between 6×His‐NPR1 and GST or GST‐tagged TuMV NIb or BSCTV V2. **(E)** Western blot showing the accumulation of NPR1‐YFP in the supernatant (cytoplasm) and pellets (nucleus) of cell lysates from *N. benthamiana* leaves coexpressing NPR1‐YFP and FLAG‐4×Myc‐V2 or FLAG‐4×Myc‐GUS separated by differential centrifugation. Histone H3 (H3) was detected using anti‐H3 antibody. The number under each lane indicates the relative intensity of the band compared with the control. T, total protein; C, cytoplasm; N, nucleus. **(F)** Relative nucleocytoplasmic ratios of NPR1 in mock, TuMV‐infected, or BSCTV‐infected transgenic *35S::NPR1‐GFP* seedlings. The data represent the average of two replicates from Figure S12. * and ** indicate *P* < 0.05 and *P* < 0.01 in Student's *t*‐test, respectively.

Encouraged by the above results, we further investigated the interaction between NPR1 and BSCTV‐encoded proteins using a BiFC assay. BSCTV encodes seven canonical proteins, namely, Rep, C2, C3, C4, CP (CP_BSCTV_), MP (MP_BSCTV_), and V2. We expressed NPR1‐YN and the seven proteins of BSCTV as YC‐tagged recombinant proteins in *N. benthamiana* leaves under the CaMV 35S promoter. Confocal microscopy observation showed that the cells coexpressing NPR1‐YN plus V2‐YC or NIb‐YC exhibited bright YFP fluorescence, and the cells coexpressing NPR1‐YN plus C4‐YC, C3‐YC or Rep‐YC exhibited a weak fluorescent signal in the nuclei. No YFP signal was observed in the cells expressing NPR1‐YN plus other YC‐tagged BSCTV proteins (Figures 5A, S7). Notably, the YFP fluorescence from the interaction between NPR1‐YN and V2‐YC was distributed both as condensates in both nuclei and cytoplasm (Figure 5B). We then confirmed the interaction between NPR1 and BSCTV V2 by co‐IP assay. The results showed that both FLAG‐4×Myc‐NIb and FLAG‐4×Myc‐V2, but not FLAG‐4×Myc‐GUS, coprecipitated with NPR1‐YFP in the co‐IP assay (Figure 5C). We further performed a pull‐down assay with purified recombinant proteins. The results showed that 6×His‐NPR1 could be pulled down by both GST‐NIb and GST‐V2, but not by GST (Figure 5D). Finally, we analyzed the interactions between BSCTV V2 and truncated or point mutants of NPR1 using BiFC. The results showed that BSCTV V2 interacts with the BTB/POZ, ANK, and TAD domains and all four NPR1 mutants in the BiFC assay (Figure S8). AMV CP interacts with the BTB/POZ domains of NPR1 (Figure 2), whereas TuMV NIb interacts with the ANK domain of NPR1 (Liu et al., 2023). Thus, BSCTV V2 may use a mechanism that differs from TuMV NIb and AMV CP to interact with NPR1.

**Figure 6:**
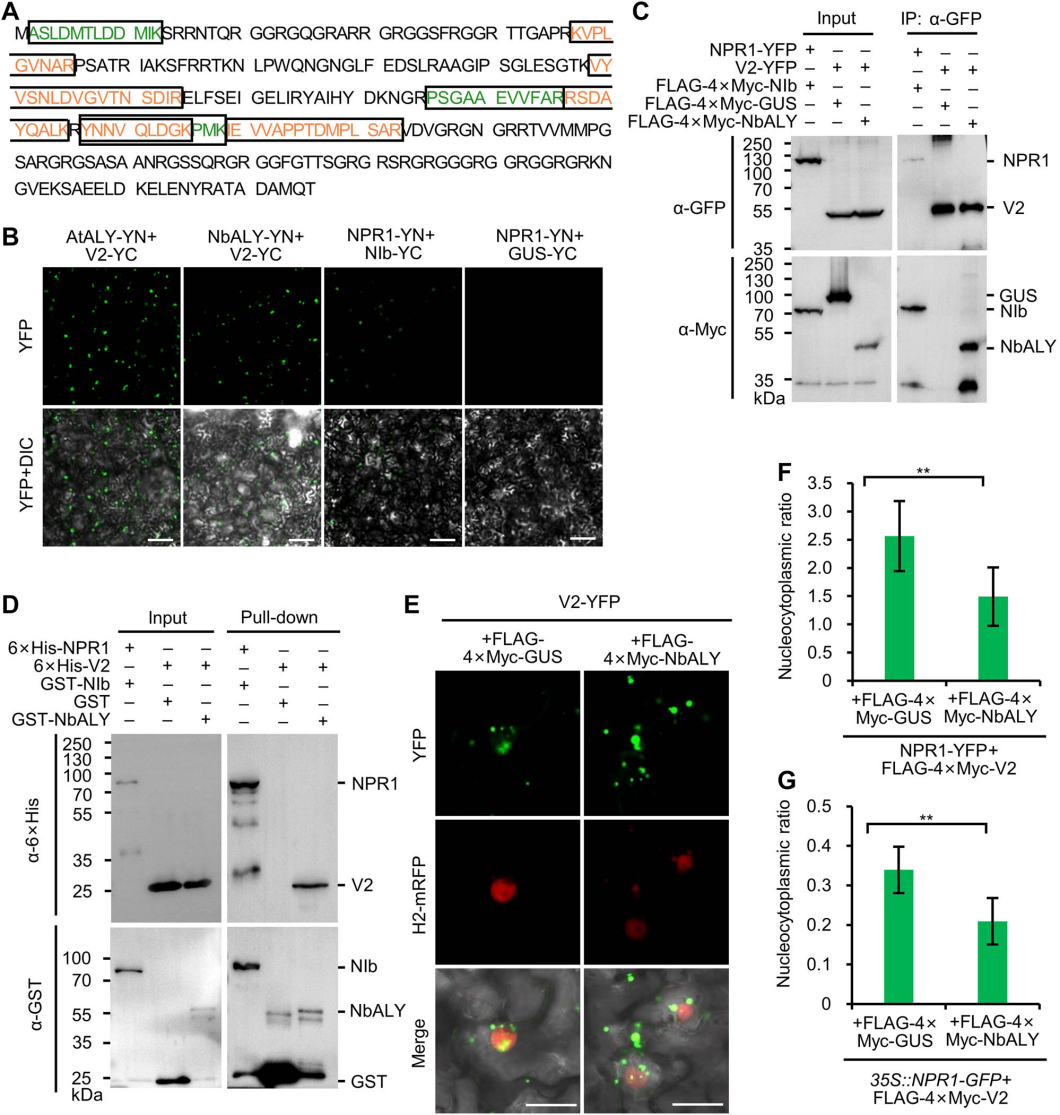
BSCTV V2 affects the nucleocytoplasmic proportion of NPR1 **(A)** Distribution of MS/MS‐identified peptides (boxed green and orange peptides) in NbALY detected in *N. benthamiana* leaves expressing V2‐YFP. **(B)** Confocal microscopy images of *N. benthamiana* epidermal cells expressing V2‐YC and AtALY‐YN or NbALY‐YC. NPR1‐YN+GUS‐YC and NPR1‐YN+NIb‐YC were used as negative and positive controls, respectively. Scale bars = 50 μm. **(C)** Co‐IP results for the interactions between V2‐YFP and FLAG‐4×Myc‐tagged GUS or NbALY. The interaction between NPR1‐YFP and FLAG‐4×Myc‐NIb was used as a positive control. All proteins were transiently expressed in *N. benthamiana* leaves by agroinfiltration, harvested at 42 hpi, affinity purified by GFP‐Trap agarose, and then analyzed with anti‐GFP or anti‐Myc antibodies. **(D)** Pull‐down assay by retention in glutathione agarose showing the interaction between BSCTV V2 and GST or GST‐NbALY. The combination of GST‐NIb and 6×His‐NPR1 was used as a positive control. All proteins were expressed in *E. coli* and GST or GST‐tagged proteins were purified using glutathione agarose. **(E)** Distribution of V2‐YFP in *N. benthamiana* epidermal cells coexpressing FLAG‐4×Myc‐GUS or FLAG‐4×Myc‐NbALY at 2 dpi. The nuclei were labeled with H2‐mRFP. Scale bars = 20 μm. **(F)** The nucleocytoplasmic ratio assessing the accumulation of NPR1‐YFP in the supernatant (cytoplasm) and pellets (nucleus) of centrifugally separated cell lysates of *N. benthamiana* leaves coexpressing NPR1‐YFP, FLAG‐4×Myc‐V2 and FLAG‐4×Myc‐GUS or FLAG‐4×Myc‐NbALY. The data represent the average of two replicates from Figure S13. **(G)** Nucleocytoplasmic ratio showing the accumulation of NPR1‐YFP in the supernatant (cytoplasm) and pellets (nucleus) of centrifugally separated cell lysates of transgenic *35S::NPR1‐GFP* leaves coexpressing FLAG‐4×Myc‐V2 and FLAG‐4×Myc‐NbALY or FLAG‐4×Myc‐GUS. The data represent the average of two replicates from Figure S14. In all panels, ** indicate *P* < 0.01, in Student's *t*‐test.

As the fluorescence pattern of the interaction between NPR1‐YN and V2‐YC is similar to that of AMV CP and NPR1, we tested whether BSCTV V2 also affects the stability of NPR1. Therefore, NPR1‐YFP was coexpressed with FLAG‐4×Myc‐tagged GUS, AMV CP, TuMV NIb, or BSCTV V2 in *N. benthamiana* leaves under the CaMV 35S promoter. Western blotting showed that the protein level of NPR1‐YFP was decreased by 80% when coexpressed with AMV CP, while the level of NPR1 was increased in the presence of TuMV NIb or BSCTV V2 (Figure S9A). To further confirm this possibility, we compared the accumulation of NPR1 in mock and BSCTV‐infected *35S::NPR1‐GFP* seedlings. The results showed that NPR1 accumulation was similar in mock and BSCTV‐infected plants (Figure S9B), confirming that BSCTV V2 did not affect the stability of NPR1. It has been reported that tomato yellow leaf curl virus (TYLCV)‐encoded V2 is a nucleocytoplasmic shuttle protein that contributes to the export of V1 protein from the nucleus to the cytoplasm for viral systemic infection (Zhao et al., 2020). Similarly, transiently expressed C‐terminal YFP‐tagged BSCTV V2 (V2‐YFP) was also localized as condensates in the nucleus and the cytoplasm of the *N. benthamiana* epidermal cells (Figure S10). We therefore hypothesized that BSCTV V2 might be able to regulate the nucleocytoplasmic distribution of NPR1. To test this, we transiently coexpressed FLAG‐4×Myc‐V2 or FLAG‐4×Myc‐GUS with NPR1‐YFP by agroinfiltration. At 2 dpi, the infiltrated leaves were harvested, and the nucleocytoplasmic distribution of NPR1‐YFP was determined by centrifugal fractionation following western blotting. We found that the nucleocytoplasmic ratio of transiently expressed NPR1‐YFP in *N. benthamiana* cells coexpressing FLAG‐4×Myc‐GUS was about 13.29 (93 to 7), which was decreased to 2.70 (73 to 27) in cells coexpressing FLAG‐4×Myc‐V2 (Figure 5E). To further confirm this possibility, we transiently expressed FLAG‐4×Myc‐V2 or FLAG‐4×Myc‐GUS in the leaves of 4‐week‐old *35S::NPR1‐GFP* seedlings and then compared the nucleocytoplasmic distribution of NPR1‐GFP by western blotting after microsomal fractionation. The results showed that the nucleocytoplasmic ratio of NPR1 was 0.96 in the leaves of *35S::NPR1‐GFP* expressing FLAG‐4×Myc‐GUS, which was decreased to 0.43 when FLAG‐4×Myc‐V2 was expressed (Figure S11). Finally, we directly compared the nucleocytoplasmic ratio of NPR1 in TuMV‐6K2mCherry‐ and BSCTV‐infected *35S::NPR1‐GFP* plants. We thus inoculated the leaves of 4‐week‐old *35S::NPR1‐GFP* seedlings with BSCTV and TuMV‐6K2mCherry, a TuMV infectious clone that contains an additional mCherry‐tagged 6K2 between P1 and HcPro cistrons (Cotton et al., 2009), and then compared the nucleocytoplasmic distribution of NPR1‐GFP in the virus‐infected leaf tissue at 10 dpi by western blotting after microsomal fractionation. The results showed that the nucleocytoplasmic ratio of NPR1 significantly increased in the TuMV‐6K2mCherry‐infected leaf tissue, whereas it significantly decreased in the BSCTV‐infected leaf tissue compared with the mock control plants (Figures 6H, S12). Taken together, these data suggest that BSCTV V2 affects the nucleocytoplasmic distribution of NPR1.

### BSCTV V2 interacts with exportin ALY to retain NPR1 in the cytoplasm

We performed MS/MS using transiently expressed V2‐YFP in *N. benthamiana* leaves to determine the host factor by which V2 affects NPR1 nucleocytoplasmic distribution. In total, 563 peptides belonging to 132 proteins were identified, including the known V2‐interacting factors Argonuate1 (AGO1) and AGO4 (Table S2). Interestingly, both the nuclear importin subunit alpha (IMPα) and Aly/REF export factor (ALY) were identified (Figure 6A), suggesting that BSCTV V2 may be a nucleocytoplasmic shuttling protein (Table S2). As we were interested in how BSCTV V2 increases the amount of NPR1 in the cytoplasm, we cloned both Arabidopsis and *N. benthamiana* ALY (*AtALY* and *NbALY*), and examined their interactions with BSCTV V2 using BiFC. The results showed that both AtALY and NbALY interacted with BSCTV V2 (Figure 6B). We further confirmed the interaction between BSCTV V2 and NbALY by co‐IP and pull‐down. The results showed that FLAG‐4×Myc‐NbALY, but not FLAG‐4×Myc‐GUS, coprecipitated with V2‐YFP (Figure 6C), and that GST‐NbALY, but not GST, was able to pull down 6×His‐V2 (Figure 6D).

We therefore hypothesized that ALY might regulate the subcellular localization of BSCTV V2 and its interacting proteins, such as NPR1. To test this hypothesis, we expressed V2‐YFP in the epidermal cells of *N. benthamiana* leaves together with FLAG‐4×Myc‐GUS or FLAG‐4×Myc‐NbALY and then compared the nucleocytoplasmic distribution of V2‐YFP at 2 dpi using confocal microscopy. The results showed that the condensed fluorescence of V2‐YFP in the cytoplasm was apparently increased in the cells coexpressing FLAG‐4×Myc‐NbALY compared with those coexpressing FLAG‐4×Myc‐GUS (Figure 6E), indicating that NbALY may regulate the nucleocytoplasmic partitioning of V2. We then analyzed the influence of ALY on the nucleocytoplasmic partitioning of NPR1 in the presence of BSCTV V2 in *N. benthamiana* and *35S::NPR1‐GFP*. We found that the nucleocytoplasmic ratio of NPR1 further decreased from 2.56 in *N. benthamiana* and 0.34 in *35S::NPR1‐GFP* cells expressing FLAG‐4×Myc‐GUS to 1.49 and 0.21 in that expressing FLAG‐4×Myc‐NbALY, respectively (Figures 6F, G, S13 and S14). Taken together, these data suggest that BSCTV V2 affects the nucleocytoplasmic distribution of NPR1 through ALY.

### Other viruses also encode proteins that interact with NPR1

**Figure 7:**
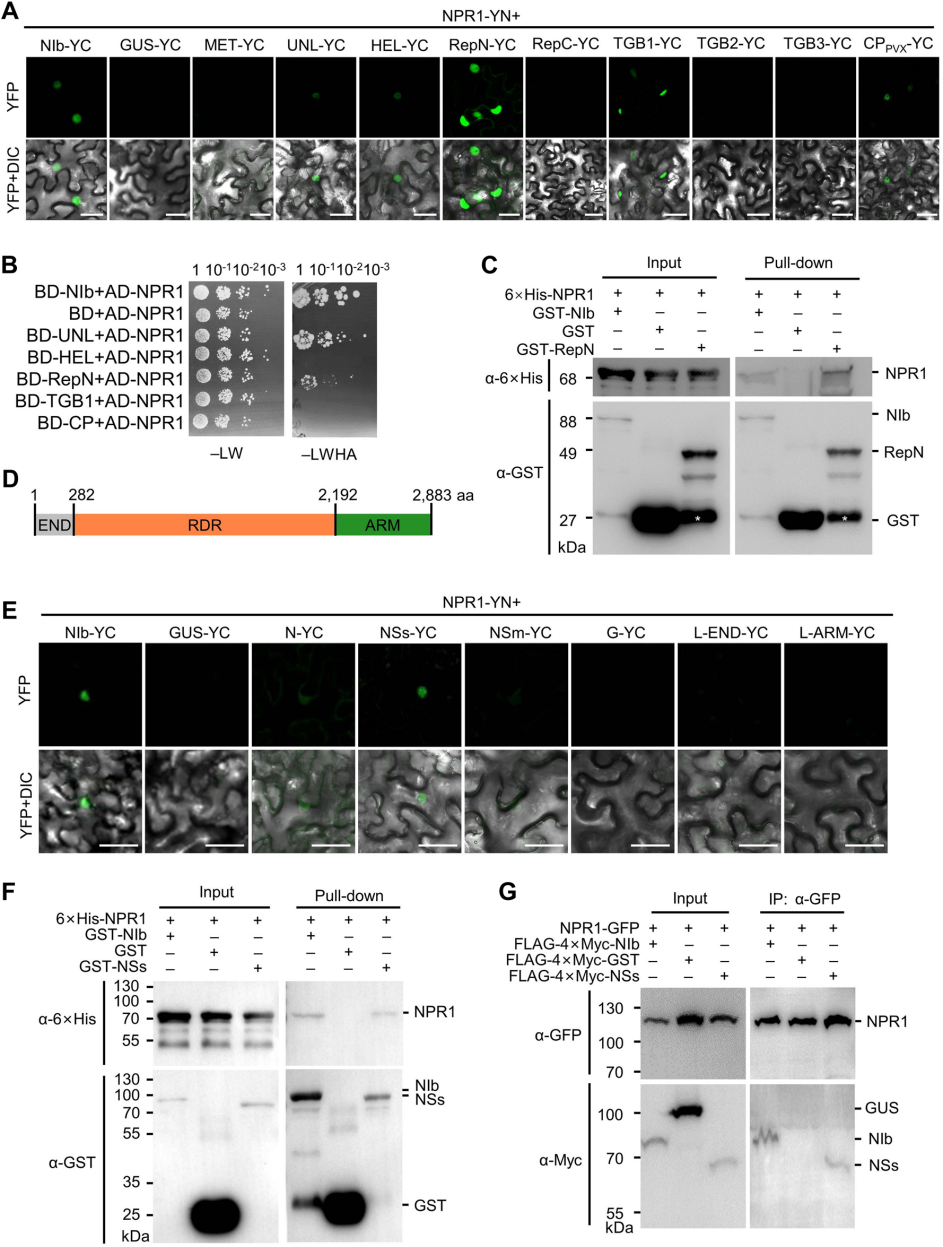
PVX and CCSV also encode proteins that interact with NPR1 **(A)** Confocal microscopy images of *N. benthamiana* epidermal cells expressing NPR1‐YN and YC‐tagged PVX‐encoded proteins at 2 dpi. MET, methyltransferase domain; UNL, unstructured domain; HEL, helicase domain; RepN, N‐terminal replicase domain; RepC, C‐terminal replicase domain. Scale bars = 50 μm. **(B)** Growth of serially diluted yeast cells expressing the indicated recombinant proteins on −LW and −LWHA medium. **(C)** Pull‐down assay by retention in glutathione agarose for the interaction between 6×His‐NPR1 and GST or GST‐tagged TuMV NIb or PVX RepN. **(D)** Illustration of CCSV L protein domains. Numbers represent the amino acid positions of domain boundaries. **(E)** Confocal microscopic photographs of the *N. benthamiana* epidermal cells expressing NPR1‐YN and YC‐tagged CCSV‐encoded proteins at 2 dpi. Scale bars = 50 μm. **(F)** Pull‐down assay by retention in glutathione agarose for the interaction between 6×His‐NPR1 and GST‐ or GST‐tagged TuMV NIb or CCSV NSs. **(G)** Co‐IP for the interactions between NPR1‐YFP and FLAG‐4×Myc‐tagged TuMV NIb, GUS, or CCSV NSs. All proteins were transiently expressed in *N. benthamiana* leaves by agroinfiltration, harvested at 48 hpi, affinity purified by GFP‐Trap agarose, and then analyzed by anti‐GFP or anti‐Myc antibodies.

To confirm that viruses from other families also interacted with NPR1, we also performed a BiFC assay between NPR1 and all PVX‐encoded proteins, namely RNA‐dependent RNA polymerase (RdRp), triple‐gene‐block protein 1 (TGB1), TGB2, TGB3, and CP (CP_PVX_). Due to the low expression level of full‐length RdRp, we divided it into five non‐overlapping fragments, namely the methyltransferase domain (MET), the unstructured domain (UNL), the helicase domain (HEL), and the N‐terminal and C‐terminal replicase domain (RepN and RepC) (Wu et al., 2019). A strong YFP signal was recorded in *N. benthamiana* epidermal cells expressing NPR1‐YN and TGB1‐YC or RepN‐YC, and a weak YFP signal was observed in the cells expressing NPR1‐YN and UNL‐YC, HEL‐YC, and CP_PVX_‐YC (Figures 7A, S15). Furthermore, the YFP signal was mainly localized in the nucleus (Figure 7A). Therefore, we performed a Y2H assay to further confirm these interactions. The results showed that the yeast cells cotransformed with AD‐NPR1 and either BD‐RepN or BD‐UNL but not BD‐TGB1 survived on the selective media (Figure 7B), suggesting that RdRp is the major viral protein that interacts with NPR1. We also performed a GST pull‐down assay and found that 6×His‐NPR1 was coprecipitated with GST‐RepN and GST‐NIb, but not with free GST (Figure 7C). Taken together, these data suggested that PVX RdRp interacts with NPR1.

Finally, we evaluated the interaction between NPR1 and proteins encoded by calla lily chlorotic spot virus (CCSV) isolated from celtuce (*Lactuca sativa* var. *augustana*) (Wu et al., 2018). CCSV is a −ssRNA virus of the genus *Orthotospovirus* in the family *Tospoviridae* of the order *Bunyavirales*, which encodes a total number of five proteins, namely L, G, NSm, N, and NSs. Based on the structure of the L protein of the severe fever with thrombocytopenia syndrome virus (SFTSV) of the same order (Wang et al., 2020), the L was further divided into three non‐overlapping fragments for better expression, namely, the N‐terminal endonuclease domain (L‐END; 1–282 aa), a middle RNA‐dependent RNA polymerase domain (L‐RDR; 283–2192 aa), and a C‐terminal arm domain with a blocker motif (L‐ARM; 2193–2883 aa) (Figure 7D). Due to low expression, L‐RDR was not tested. The BiFC assay showed that a bright fluorescence signal was observed in the epidermal cells of *N. benthamiana* expressing NPR1‐YN and NSs‐YC, and a weak fluorescence signal was observed in the epidermal cells of *N. benthamiana* expressing NPR1‐YN and N‐YC (Figures 7E, S16). As the Y2H assay indicated that the NSs had strong self‐activating activity, we performed GST pull‐down and co‐IP assays to confirm the interaction. The results showed that 6×His‐NPR1 was coprecipitated with GST‐NSs and GST‐NIb, but not GST, in the GST pull‐down assay, while FLAG‐4×Myc‐NIb and FLAG‐4×Myc‐NSs, but not FLAG‐4×Myc‐GUS, were coprecipitated with NPR1‐YFP (Figure 7F, G). Taken together, these data suggested that NSs is the viral effector of CCSV that interacts with NPR1.

## DISCUSSION

It is already known that the induction of plant innate immunity stimulates SA signaling, which leads to the expression of various defense‐related genes, a phenomenon known as systemic acquired resistance (SAR) (Fu and Dong, 2013). However, the function of SA signaling in the compatible plant–virus interaction, in which viruses spread systemically and cause diseases, is not well understood. We recently found that the SA signaling pathway plays an important inhibitory role in the compatible infection by TuMV in Arabidopsis, and that approximately 90% of PR gene expression is dependent on this pathway (Liu et al., 2023). Here, we further show that knockout of *NPR1* increases the susceptibility of Arabidopsis to AMV and BSCTV, whereas overexpression of *NPR1* enhances the resistance of Arabidopsis to these two viruses (Figure 1). Moreover, direct spraying of SA also increased the resistance of *N. benthamiana* to PVX and BSCTV (Figure 1). These data suggest that SA signaling may play a broad‐spectrum antiviral role in the compatible plant–virus interaction. Interestingly, the SA spray experiment showed that SA could inhibit PVX proliferation throughout the testing period, while it only displayed an inhibitory effect during the early infection stage of BSCTV. These differences suggest that the antiviral effects of the SA signaling pathway on different viruses are markedly different, which may be caused by the different replication mechanisms, movement strategies, and/or the ability to inhibit the SA signaling pathway.

Given the importance of the SA signaling pathway in plant immunity, it is not surprising to find that many pathogens have evolved various strategies to disrupt this pathway, such as producing the jasmonate acid (JA) structural mimic coronatine (COR), encoding the salicylate hydroxylase NahG, which degrades SA to catechol, binding the SA receptor NPR1, and directly inhibiting the transcription of defense‐related genes via targeting transcription factor TGA (van Wees and Glazebrook, 2003; Zheng et al., 2012; Sun et al., 2018; Qi et al., 2022; Ghosh and Chakraborty, 2023). Among these strategies to inhibit SA signaling, targeting NPR1 seems to be a common strategy adopted by different phytopathogens. In the case of viruses, TuMV NIb interacts with the central ANK domain and inhibits NPR1 SUMOylation, whereas RSV p2 interacts with the C‐terminal TAD domain to induce NPR1 degradation via Cullin3 (CUL3), the key component of Cullin3–RING (CRL3) E3 ligase (Liu et al., 2023; Zhang et al., 2023). In this study, we found that four other plant viruses also encode one or more proteins that interact with NPR1, highlighting this plant factor as an important target of plant viruses in general. Among all these viruses, TuMV, AMV, and PVX belong to three different orders of the six +ssRNA viral orders that include plant‐infecting families, namely, *Patatavirales*, *Martellivirales*, and *Tymovirales*, while BSCTV and CCSV are ssDNA and –ssRNA viruses, respectively. These data strongly suggest that targeting NPR1 may be a general mechanism used by most, if not all, plant viruses to suppress this pathway. For example, almost all plant viruses encode a protein that inhibits RNA silencing, a versatile antiviral defense mechanism in eukaryotes (Wu et al., 2010).

However, the mechanism of NPR1 inactivation varies depending on the virus and the interacting protein. Recently, we reported that TuMV NIb targets the ANK domain of NPR1 in the nucleus and inhibits its SUMOylation (Liu et al., 2023). Here, we found that AMV CP targets the N‐terminal BTB/POZ domain of NPR1 (Figure 2) and destabilizes the protein by recruiting the Skp1 of the SCF E3 ligase (Figures 3, 4). BSCTV V2, on the other hand, can interact with all three domains of NPR1 and all tested mutants in the BiFC assay (Figure S8) and may act by modifying the nucleocytoplasmic location of NPR1 through its interaction with ALY (Figures 5, 6). These viral proteins do not share any sequence similarity and have completely different functions in the proliferation of their respective viruses. NIb is the RNA‐dependent RNA polymerase of potyviruses, while p2 is the suppressor of host RNA silencing (Xiong et al., 2009; Shen et al., 2020b). AMV CP is required not only for viral genome encapsulation but also for activation of viral replication (Reichert et al., 2007). It also interacts with and interferes with the transcriptional activity of a basic helix–loop–helix (bHLH) family transcription factor (ILR3) in the nucleus to regulate plant defense responses (Aparicio and Pallás, 2017). BSCTV V2 is a nucleocytoplasmic shuttling protein that is required for the nuclear export of V1 protein and suppresses host RNA silencing possibly by targeting AGO proteins (Luna et al., 2017, 2020; Zhao et al., 2020). These data strongly support that these viral proteins have evolved independently to target NPR1, highlighting the importance of the SA signaling pathway in the virus–host interaction.

In conclusion, we report that four different viruses all encode at least one protein that targets NPR1 and elucidate the molecular mechanisms by which AMV CP and BSCTV V2 inhibit the function of the NPR1 protein. These results further enrich our understanding of the SA signaling pathway and viral strategies to inhibit NPR1.

## MATERIALS AND METHODS

### Plant materials, growth condition, and virus inoculation

The *npr1‐0*, *npr1‐1*, and transgenic Arabidopsis *35S::NPR1‐GFP* (line 4) have been described previously (Liu et al., 2023). *N. benthamiana* plants were grown in a climate chamber at 23°C under a 16 h/8 h (light/dark) photoperiod, while *A. thaliana* seedlings were grown in a growth chamber at 23°C under a 12 h/12 h photoperiod. Mechanical inoculation was performed as described previously (Liu et al., 2023).

### Plasmid construction

The full‐length coding sequences of AMV *1a*, *2a*, *MP*, and *CP* were amplified from the AMV infectious cDNA clone pCB301‐AMV RNA1, RNA2, and RNA3, respectively (Che et al., 2020). The full‐length coding sequences of BSCTV *C4*, *C3*, *C2*, *Rep*, *CP*, *MP*, and *V2* were cloned from the BSCTV infectious DNA clone pCambia1300‐BSCTV (Chen et al., 2010). The full‐length coding sequences of *NbSkp1.1* (KP017273.1), *NbSkp1.2* (KP017274.1), *NbSkp1.3* (KP017275.1), *AtASK1* (AT1G75950), *AtASK2* (AT5G42190), *NbALY* (AM167906.1), and *AtALY* (AT5G37720) were cloned from wild‐type Arabidopsis and *N. benthamiana*. The coding sequences of CCSV *L*, *G*, *NSm*, *N*, and *NSs* were amplified from the reverse transcribed cDNA from the CCSV‐infected by celtuce sample (Wu et al., 2018). All polymerase chain reactions (PCR) were performed using the Phanta Flash Master Mix (Vazyme, Nanjing, China) with the primers listed in Table S3. Amplified fragments were inserted into Gateway‐compatible entry vector pDONR‐207‐SapI using the ClonExpress II (Vazyme) and then transferred into the Gateway‐compatible binary plasmids using the Gateway™ LR Clonase™ II Enzyme Mix (Thermal Fisher Scientific, Shanghai, China). The Gateway‐compatible vectors pEarleyGate201‐YN and pEarleyGate202‐YC were used for the BiFC assay (Lu et al., 2010), pEarleyGate‐101, pGWB‐554, and pBA‐FLAG‐4×Myc‐DC were used for transient protein expression (Earley et al., 2006; Nakagawa et al., 2007; Zhu et al., 2011), pGBKT7‐DEST and pGADT7‐DEST were used for the Y2H assay (Lu et al., 2010); pET‐32a (+) (Merck Millipore, Beijing, China) and pGEX‐4T‐1 (Cytiva, Marlborough, MA, USA) were used for protein expression in *Escherichia coli*. All plasmids were verified by Sanger sequencing. The plasmids carrying wild‐type or mutant *NPR1* (At1g64280) and PVX‐encoded truncated *RdRp* fragments, *TGB1*, *TGB2*, *TGB3*, and *CP* were characterized previously (Wu et al., 2019; Liu et al., 2023).

### Agroinfiltration

*Agrobacterium rhizogenes* strain GV3101 carrying the target plasmid was cultured in liquid Luria–Bertani medium supplemented with the appropriate antibiotics at 28°C, pelleted by low‐speed centrifugation at room temperature, resuspended in infiltration buffer (10 mM MgCl_2_; 100 μM acetosyringone; 10 mM 2‐morpholinoethanesulfonic acid, pH 5.6), and left at room temperature for 3 h. The *Agrobacterium* solution was infiltrated into *N. benthamiana* or Arabidopsis leaves at a final optical density at 600 nm (OD_600_) of 0.4 for transient recombinant protein expression, and at OD_600_ 0.2 for virus inoculation.

### Nucleic acid extraction, reverse transcription, and qPCR

Total RNA was extracted using the Eastep® Super Total RNA Extraction Kit (Promega, Beijing, China) according to the supplied protocol. Total DNA was isolated using the One‐step Plant Genomic DNA Rapid Extraction Reagent (Bioteke, Beijing, China) according to the provided protocol. First‐strand cDNA was synthesized using the HiScript® 1st Strand cDNA Synthesis (+gDNA wiper) Kit (Vazyme). qPCR was performed in a 20 μl volume system with a CFX Connect™ Real‐Time System (Bio‐Rad, California, USA). The genomic RNAs of AMV and PVX were determined by amplification of a 135 bp fragment of the *CP* gene and a 190 bp fragment of the *CP* gene. The genomic DNA of BSCTV was determined by amplification of a 77 bp fragment of the C3 gene. The *A. thaliana ACTIN II* (AT3G18780) and the *N. benthamiana ACTIN* (AY179605) were used as internal controls. All experiments were repeated three times, and the primers are listed in Table S3.

### Chemical treatment

SA was dissolved in distilled water and sprayed on *N. benthamiana* leaves at a final concentration of 0.5 mM. Cycloheximide (CHX; Sigma‐Aldrich, Beijing, China) was dissolved in distilled water, while 3‐methyladenine (3‐MA; Sigma‐Aldrich) and Z‐Leu‐Leu‐Leu‐al (MG132; Sigma‐Aldrich) were dissolved in dimethyl sulfoxide (DMSO). CHX, 3‐MA, and MG‐132 were infiltrated into leaves using a 1‐mL needleless syringe at final concentrations of 100 μM, 10 mM, and 100 μM, respectively.

### Confocal microscopy

Confocal microscopy was performed as previously described (Liu et al., 2023). Briefly, *N. benthamiana* leaves expressing target proteins were dissected and observed using a Leica TCS SP8 laser scanning confocal microscope (Leica, Germany). The YFP and mRFP were activated with 514 nm and 568 nm lasers, respectively. The sequential model was used when more than one protein was imaged simultaneously.

### SDS‐PAGE and Western blotting

Proteins were separated in 12% polyacrylamide gels and transferred to a polyvinylidene fluoride (PVDF) membrane using a Trans‐Blot Turbo Transfer System. After blocking in Tris‐buffered saline with Tween 20 (TBST) containing 5% non‐fat milk powder overnight at 4°C or for 1 h at room temperature, the PVDF membrane was incubated in TBST containing 5% non‐fat milk and appropriate primary antibodies for 1 h at room temperature. The PVDF membrane was washed eight times with TBST and incubated again in TBST containing 5% non‐fat milk and an appropriate secondary antibody. Finally, the PVDF membrane was washed eight times with TBST and visualized using a Tanon 5200CE chemiluminescence imaging system after soaking in Immobilon Western chemiluminescent horseradish peroxidase (HRP) substrate solution (Merck Millipore). In each experiment, a parallel gel was stained with Coomassie Brilliant Blue as a loading control.

Rabbit polyclonal anti‐GFP N‐terminal antibodies (Sigma‒Aldrich, Shanghai, China), anti‐Myc antibodies (Abcam, Shanghai, China), and anti‐Histone H3 (Abcam) were used at 1:5,000, 1:5,000, and 1: 2,500 dilutions, respectively. Mouse monoclonal anti‐GFP C‐terminal antibody (Roche, Shanghai, China), and anti‐mRFP antibody (Proteintech, Wuhan, Hubei, China) were used at a 1:1,000 and 1:5,000 dilution, respectively. Mouse monoclonal anti‐His (Santa Cruz Biotechnology, Shanghai, China) and anti‐GST (Santa Cruz Biotechnology) were used at a 1:2,000 dilution. The mouse monoclonal antibody against AMV CP has been reported previously and was used at a 1:5,000 dilution (Zhan et al., 2023), while rabbit polyclonal anti‐NPR1 was used at a 1:1,000 dilution (Liu et al., 2023). HRP‐conjugated anti‐rabbit IgG secondary antibodies (Sigma‐Aldrich) and HRP‐conjugated anti‐mouse IgG secondary antibodies (Sigma‐Aldrich) were used at a 1:5,000 dilution. Bands were quantified using the Gel‐Pro Analyzer 3.0.

### Nucleocytoplasmic fractionation

Nucleocytoplasmic fractionation assays were performed as previously described (Liu et al., 2023). Briefly, plant materials were ground to fine powder in liquid nitrogen and then resuspended in extract buffer (10 mM Tris‐HCl pH 8.0; 0.4 M sucrose, 10 mM MgCl_2_, 5 mM *β*‐mercaptoethanol, 0.1 mM phenylmethylsulfonyl fluoride, and 0.5 tablet of complete protease inhibitor) at 1:2 (weight to volume). The cell lysate was filtered through Miracloth and then centrifuged at 4,000 × *g* for 20 min at 4°C. The supernatant was transferred to a new tube, and pellets containing nuclei were resuspended in an equal volume of extraction buffer containing 1% Nonidet P40 (NP40). Equal volumes of the supernatant and nuclear lysate were used for subsequent SDS‐PAGE and western blotting.

### Co‐IP and mass spectrometry analysis

*N. benthamiana* leaf samples (2 g) were ground to fine powder in liquid nitrogen, then resuspended in 8 mL IP buffer (50 mM Tris‐HCl pH7.5; 150 mM NaCl; 0.1% TritonX‐100; 0.1% NP‐40; 5 mM EDTA; 2 mM DTT; 5% glycerin; 1 mM PMSF; one tablet of complete protease inhibitor [Roche, Shanghai, China]) and placed on ice for 20 min. After filtration through Miracloth (Merck Millipore), the solution was subjected to sonication for 20 min (10% power, 10 s sonication with an interval of 1 min). The mixture was then centrifuged at 6,000 × *g* at 4°C for 20 min, after which the soluble proteins were added with 30 μL of Chromotek GFP‐Trap (Proteintech) or anti‐FLAG M2 affinity gel (Sigma‒Aldrich) and incubated at 4°C for 3 h. The beads were collected by centrifugation at 1,000 × *g* at 4°C for 5 min, washed five times with IP buffer, and frozen at −80°C until use, or used directly for western blotting or sent to Bioprofile Technology (Shanghai) for MS/MS. In brief, the proteins were digested with trypsin (Promega) and analyzed using an Easy nLC 1200 mass spectrometer (Thermo Fisher Scientific, Shanghai, China). Data were searched against the *N. benthamiana* uniport (accessed on April 10, 2023) using MaxQuant 2.0.1.0. Peptides present only in the sample expressing the target protein, but not in the sample expressing free YFP, were recognized as interacting candidates.

### Yeast‐two‐hybrid assay

Plasmids were transformed into *Saccharomyces cerevisiae* strain Y2HGold using the Yeast Transformation Kit (Coolaber, Beijing, China). All transformants were grown for 3 d at 30°C on a synthetic defined (SD) medium lacking leucine and tryptophan (−LW) and then transferred to an SD medium lacking leucine, tryptophan, histidine, and adenine (−LWHA).

### Protein expression in bacteria and GST pull‐down

Recombinant proteins were expressed in *E. coli* BL21(DE3) cells with 0.1 mM or 0.5 mM isopropyl‐*β*‐D‐thiogalactoside (IPTG) at 16°C overnight. Bacteria were harvested by centrifugation at 6,000 rpm for 10 min at 4°C, and then resuspended in 10 mL lysis buffer (for Trx‐His fusion proteins: 20 mM Tris‐HCl, pH8.0; 500 mM NaCl; 20 mM imidazole; for GST and GST‐tagged proteins: 20 mM Tris‐HCl pH8.0; 500 mM NaCl; 1 mM *β*‐mercaptoethanol). The bacterial suspension was sonicated for 40 min on ice with 10% power intensity (1 min break after every 10 s of sonication) until the suspension became clear. GST‐labeled and 6×His‐labeled proteins were purified with glutathione agarose (Thermo Fisher Scientific) and Ni‐NTA agarose (Thermo Fisher Scientific), respectively. Purified GST‐tagged and 6×His‐tagged proteins were incubated with glutathione agarose beads at 4°C for 3 h. The beads were washed five times with lysis buffer, then frozen at −80°C until use or used directly for SDS‐PAGE and western blotting.

### Statistical analysis

All statistical analyses were performed using Student's two‐tailed *t*‐test in Office Excel. All data are presented as the mean ± *SD*.

## CONFLICTS OF INTEREST

The authors declare no conflicts of interest.

## AUTHOR CONTRIBUTIONS

X.J., Y.Y., and Y.L. performed the experiments and analyzed data. Y.W. produced the polyclonal antibodies against AMV CP. B.R., W.J., and X.W. contributed new reagents/analytic tools; J.A.G. revised the manuscript. X.C. and X.W. designed the experiments, supervised the study, and wrote and revised the paper. All authors read and approved its content.

## Supporting information

Additional Supporting Information may be found online in the supporting information tab for this article: http://onlinelibrary.wiley.com/doi/10.1111/jipb.13866/suppinfo

**Figure S1.** Immunoblots for the expression of recombinant proteins in Figure 2A
**Figure S2.** Immunoblots for the expression of recombinant proteins Figure 2E
**Figure S3.** Co‐IP assay for the interaction between AMV CP and the ANK (A) and TAD (B) domains of NPR1
**Figure S4.** Western blot for the expression of recombinant proteins in Figure 3A
**Figure S5.** BiFC assay for the interaction between AMV CP and Arabidopsis ASK1 and ASK2 in *N. benthamiana* epidermal cells
**Figure S6.** Arabidopsis ASK caused the degradation of NPR1
**Figure S7.** Immunoblots for the expression of recombinant proteins in Figure 5A
**Figure S8.** BiFC assay for the interactions between V2‐YC and YN‐tagged truncated or point mutants of NPR1 in *N. benthamiana* epidermal cells
**Figure S9.** V2 does not affect NPR1 stability
**Figure S10.** Confocal microscopic photographs of *N. benthamiana* epidermal cells expressing V2‐YFP
**Figure S11.** BSCTV V2 affects NPR1 nucleocytoplasmic distribution in Arabidopsis
**Figure S12.** ALY increases the effects of V2 on NPR1 nucleocytoplasmic distribution
**Figure S13.** ALY increases the effects of V2 on NPR1 nucleocytoplasmic distribution in Arabidopsis
**Figure S14.** Western blotting showing the nucleocytoplasmic distribution of NPR1 in *35S::NPR1‐GFP* at the presence of BSCTV and TuMV‐GFP
**Figure S15.** Immunoblots for the expression of recombinant proteins Figure 7A
**Figure S16.** Immunoblots for the expression of recombinant proteins Figure 7E

**Table S1.** Peptides affinity purified by tandem mass spectrometry (MS/MS) using transiently coexpressed FLAG‐4×Myc‐CP_AMV_ and NPR1‐YFP in *N. benthamiana* leaves

**Table S2.** Peptides identified by tandem mass spectrometry (MS/MS) using transiently expressed V2‐YFP in *N. benthamiana* leaves

**Table S3.** Primers used in this study
