# Supplementary material for: Plant viruses convergently target NPR1 with various strategies to suppress salicylic acid‐mediated antiviral immunity
Source: J Integr Plant Biol. 2025 Feb 21;67(5):1395–412. doi: 10.1111/jipb.13866 (PMC12060747; doi:10.1111/jipb.13866)
Supplement: Supplementary file 1 — Figure S1. Immunoblots for the expression of recombinant proteins in Figure 2A Figure S2. Immunoblots for the expression of recombinant proteins Figure 2E Figure S3. Co‐IP assay for the interaction between AMV CP and the ANK (A) and TAD (B) domains of NPR1 Figure S4. Western blot for the expression of recombinant proteins in Figure 3A Figure S5. BiFC assay for the interaction between AMV CP and Arabidopsis ASK1 and ASK2 in N. benthamiana epidermal cells Figure S6. Arabidopsis ASK caused the degradation of NPR1 Figure S7. Immunoblots for the expression of recombinant proteins in Figure 5A Figure S8. BiFC assay for the interactions between V2‐YC and YN‐tagged truncated or point mutants of NPR1 in N. benthamiana epidermal cells Figure S9. V2 does not affect NPR1 stability Figure S10. Confocal microscopic photographs of N. benthamiana epidermal cells expressing V2‐YFP Figure S11. BSCTV V2 affects NPR1 nucleocytoplasmic distribution in Arabidopsis Figure S12. ALY increases the effects of V2 on NPR1 nucleocytoplasmic distribution Figure S13. ALY increases the effects of V2 on NPR1 nucleocytoplasmic distribution in Arabidopsis Figure S14. Western blotting showing the nucleocytoplasmic distribution of NPR1 in 35S::NPR1‐GFP at the presence of BSCTV and TuMV‐GFP Figure S15. Immunoblots for the expression of recombinant proteins Figure 7A Figure S16. Immunoblots for the expression of recombinant proteins Figure 7E [file JIPB-67-1395-s001.doc]

**Supplemental Figures**

**
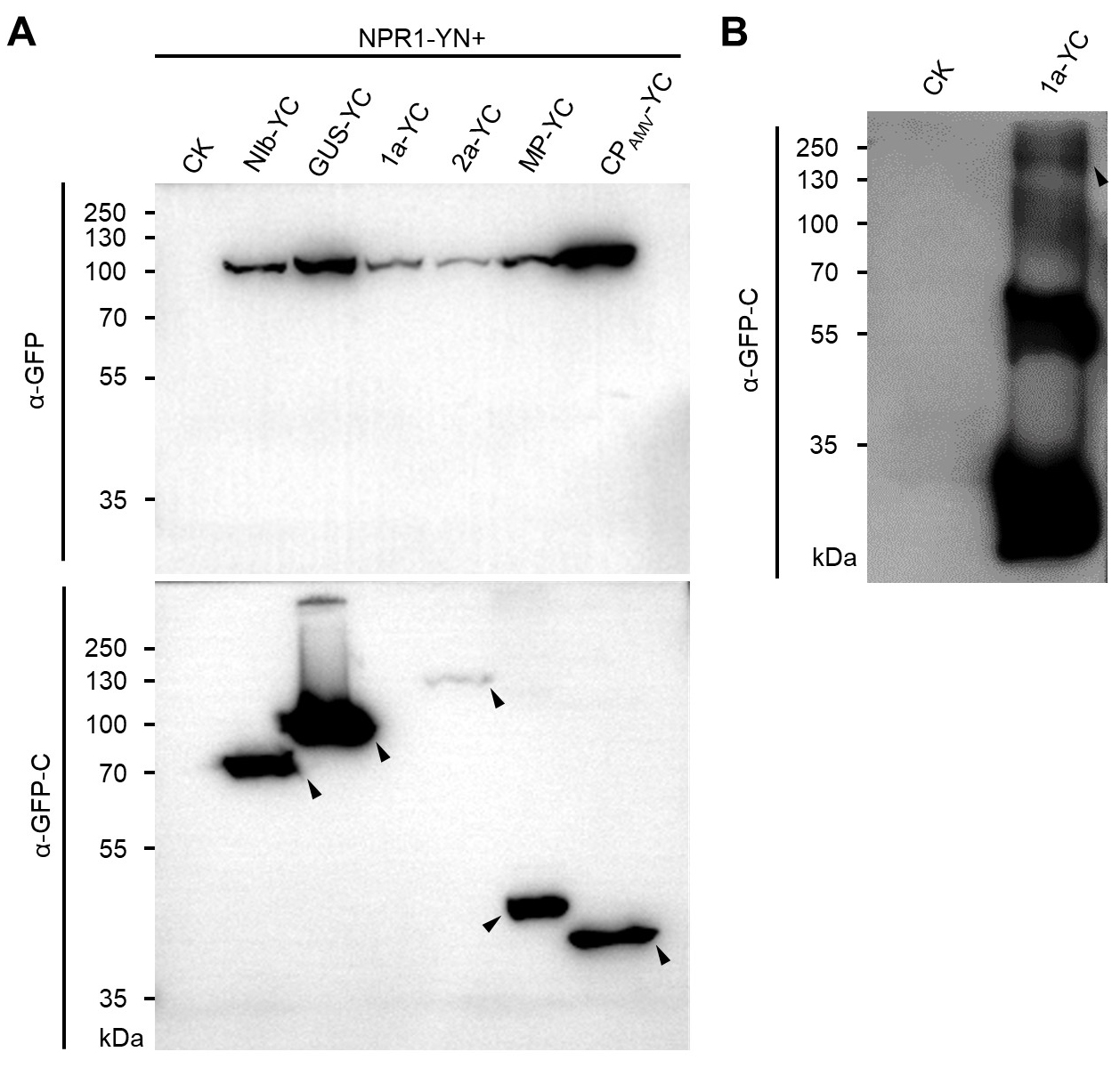
**

**Figure S1.** **Immunoblots for the expression of** **recombinant proteins in Fig. 2A.**

The expression of NPR1-YN was detected by polyclonal antibodies against the N-terminal domain of GFP (α-GFP) and YC-tagged NIb, GUS, 1a, 2a, MP, and CP_AMV_ were detected by monoclonal antibody against the C-terminal domain of GFP (α-GFP-C). (B) Immunoprecipitation and Western blot for the expression of 1a-YC. Due to low expression levels, 1a-YC was affinity purified by anti-FLAG M2 affinity gel and then analyzed by anti-GFP-C. The position of recombinant proteins isindicated by black arrowheads.

**
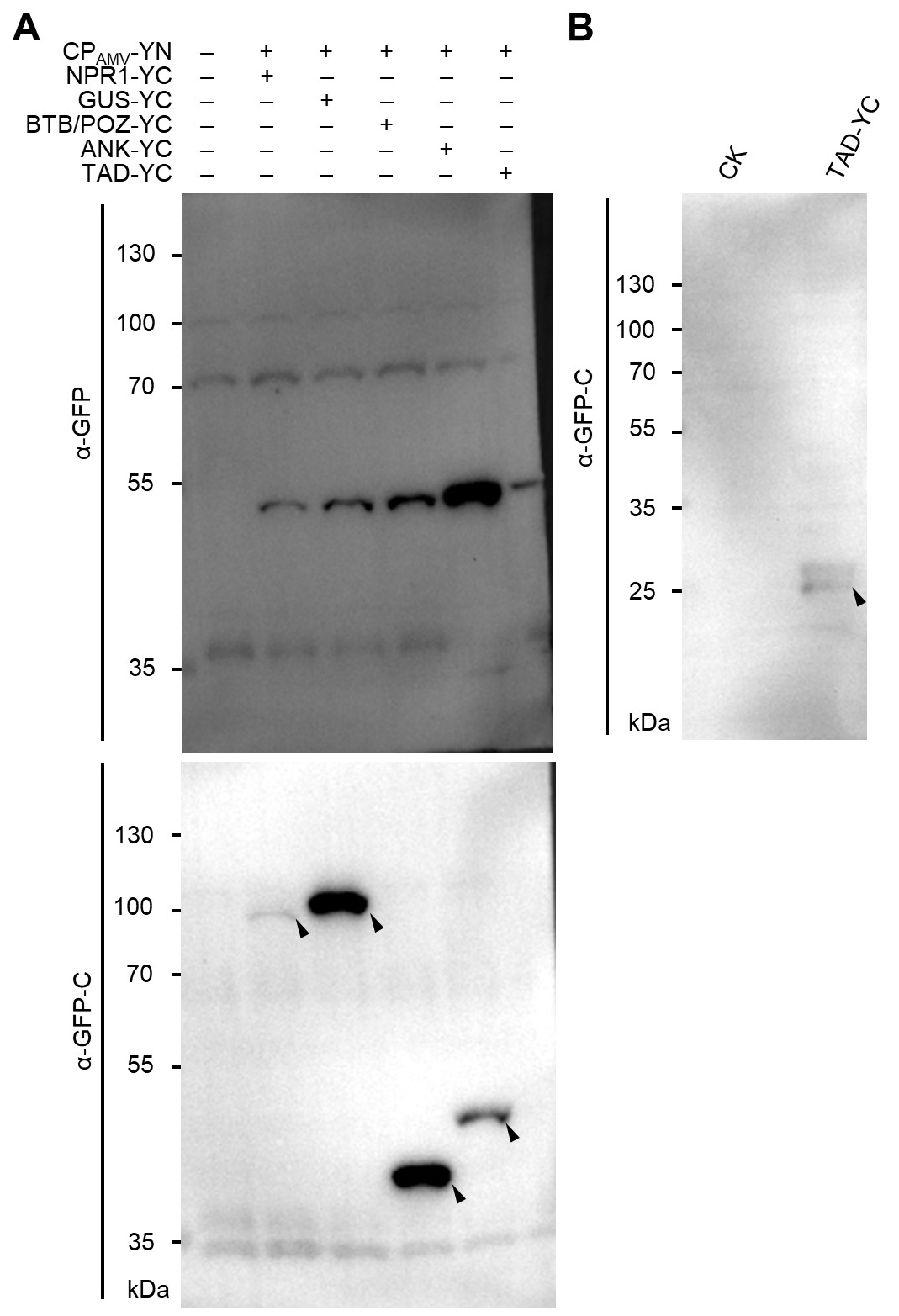
**

**Figure S2.** **Immunoblots for the expression of recombinant proteins Fig. 2E.**

(A) The expression of NCP_AMV_-YN was detected by α-GFP and YC-tagged NPR1, GUS, BTB/POZ, ANK, and TAD were detected by α-GFP-C. (B) Immunoprecipitation and Western blot for the expression of TAD-YC. TAD-YC was affinity purified by anti-FLAG M2 affinity gel and then analyzed by anti-GFP-C. The position of recombinant proteins are indicated by black arrow-heads.


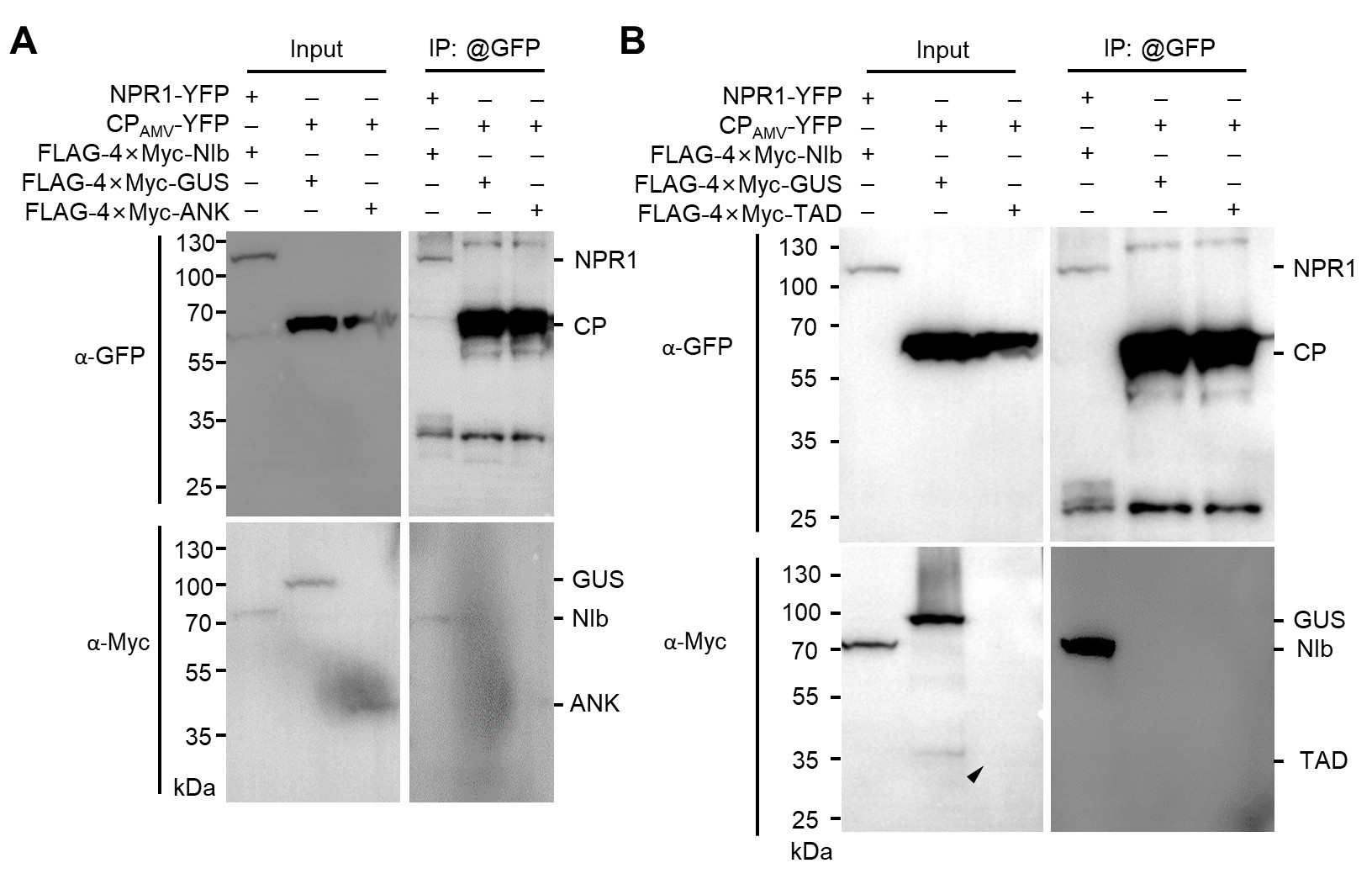


**Figure S3.** **CoIP assay for the interaction between AMV CP and the ANK (A) and TAD (B) domains of NPR1.**

Proteins were expressed by agroinfiltration in *N. benthamiana* leaves and affinity purified by GFP-Trap agarose at 42 hpi. NPR1-YFP and FLAG-4×Myc-tagged proteins were detected using α-GFP and α-Myc, respectively.


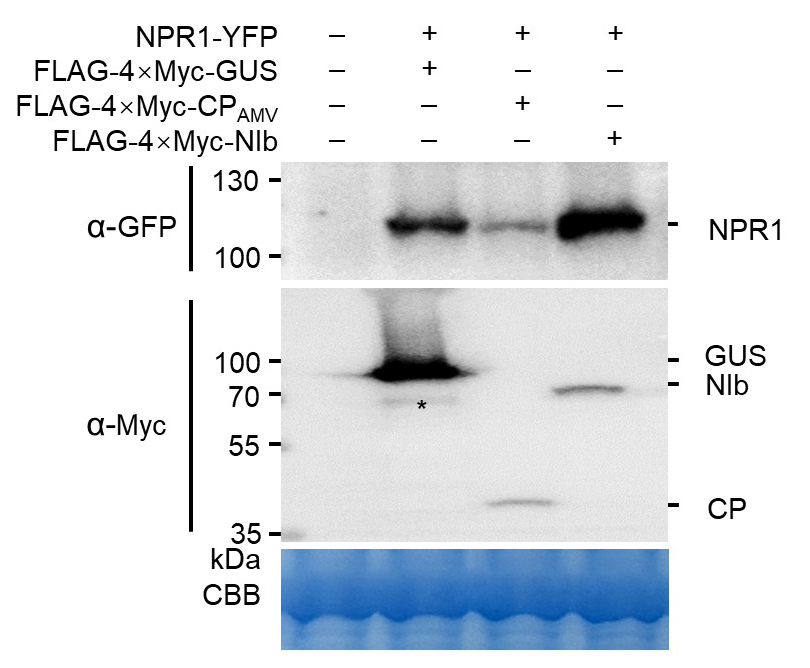


**Figure S4.** **Western blot for the expression of recombinant proteins in Fig. 3A.**

NPR1-YFP was detected by α-GFP, and FLAG-4×Myc-tagged GUS, NIb and CP_AMV_ were detected by α-Myc. CBB, Coomassie Brilliant Blue stain for loading control.


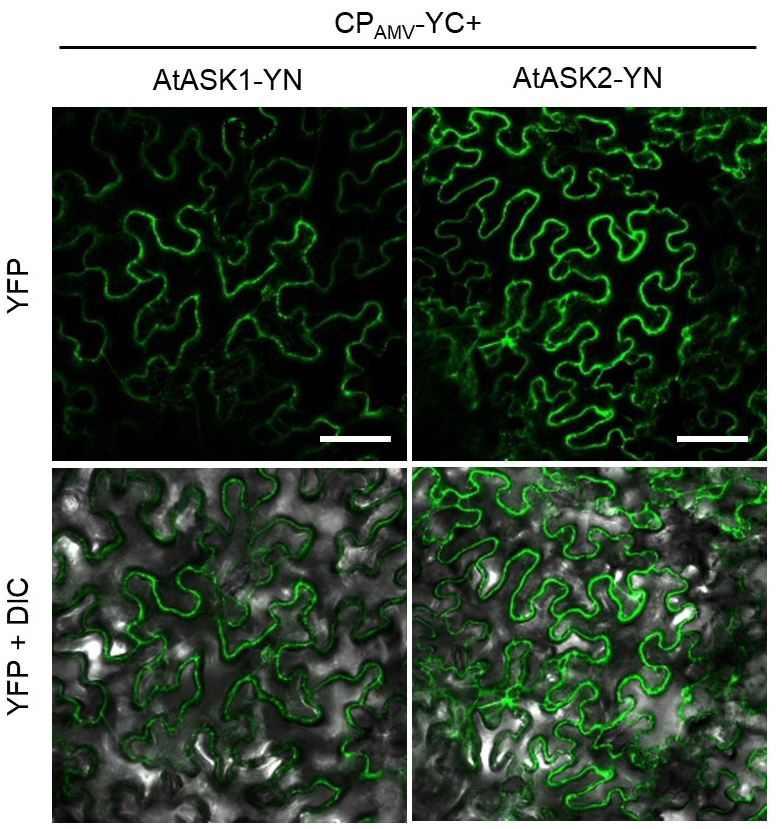


**Figure S5. BiFC assay for the interaction between AMV CP and Arabidopsis ASK1 and ASK2 in *N. benthamiana* epidermal cells.**

Proteins were expressed by agroinfiltration, and photographs were taken at 2 dpi with identical settings; DIC, differential interference contrast channel; scale bars = 50 μm.


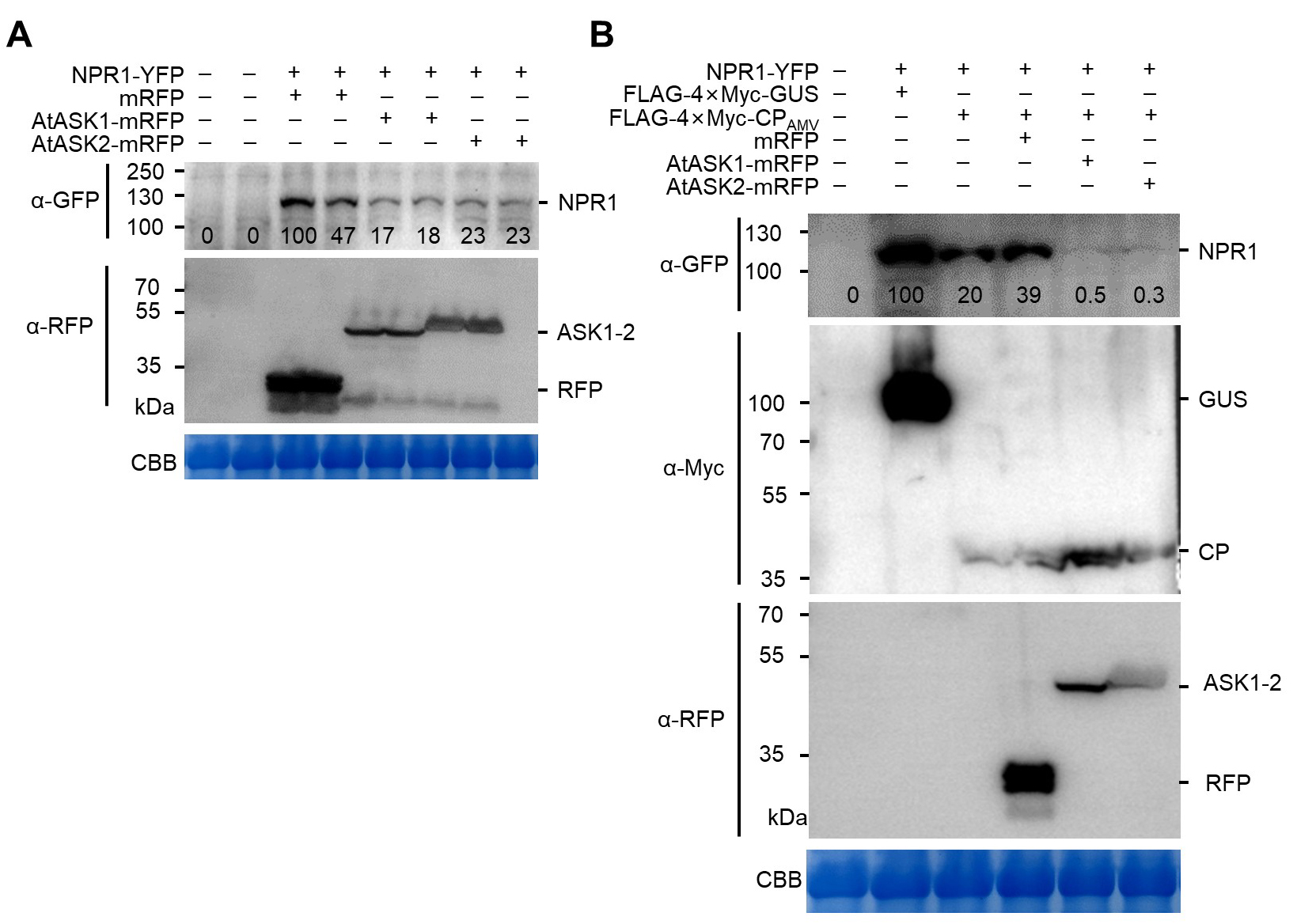


**Figure S6.** **Arabidopsis ASK caused the degradation of NPR1.**

(A) Immunoblot for the accumulation of NPR1-YFP in the *N. benthamiana* cells coexpressing mRFP or mRFP-tagged AtASK1, or AtASK2. NPR1-YFP was detected by α-GFP and mRFP-tagged recombinant proteins were detected by polyclonal antibodies against mRFP (α-mRFP). The number under each lane represents the relative intensity of the band to the control. (B) Immunoblot for the accumulation of NPR1-YFP in the *N. benthamiana* cells coexpressing FLAG-4×Myc-CP_AMV_ and mRFP-tagged AtASK1, or AtASK2. The number under each lane represents the relative intensity of the band to the control. CBB, Coomassie Brilliant Blue stain for loading control.


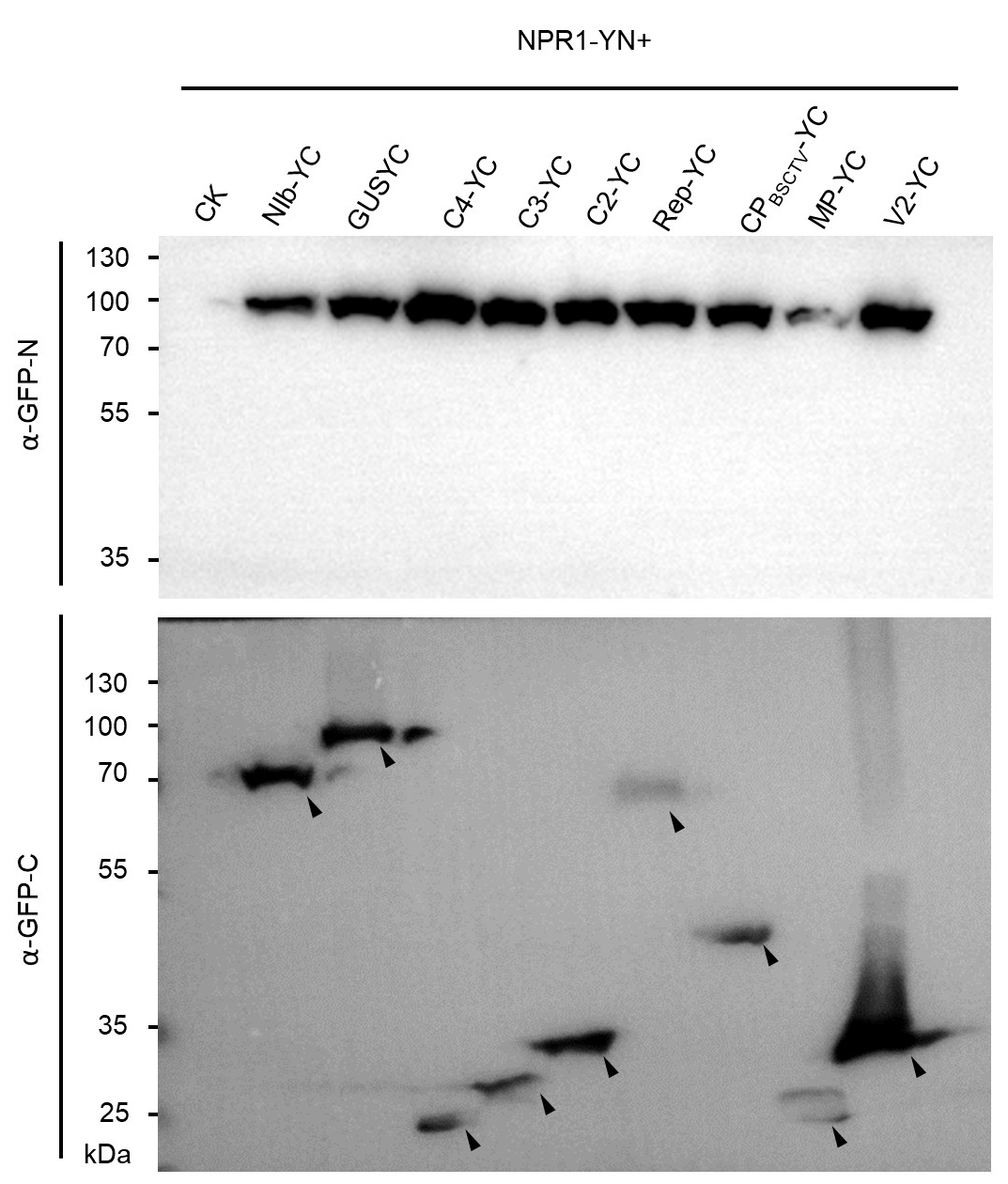


**Figure S7.** **Immunoblots for the expression of recombinant proteins in Fig. 5A.**

The expression of NPR1-YN was detected by α-GFP and YC-tagged NIb, GUS, C4, C3, C2, Rep, CP_BSCTV_, MP, and V2 were detected by α-GFP-C. The position of C4-YC, C3-YC, C2-YC, Rep-YC, CP-YC, MP-YC, and V2-YC are indicated by black arrow-heads.

**
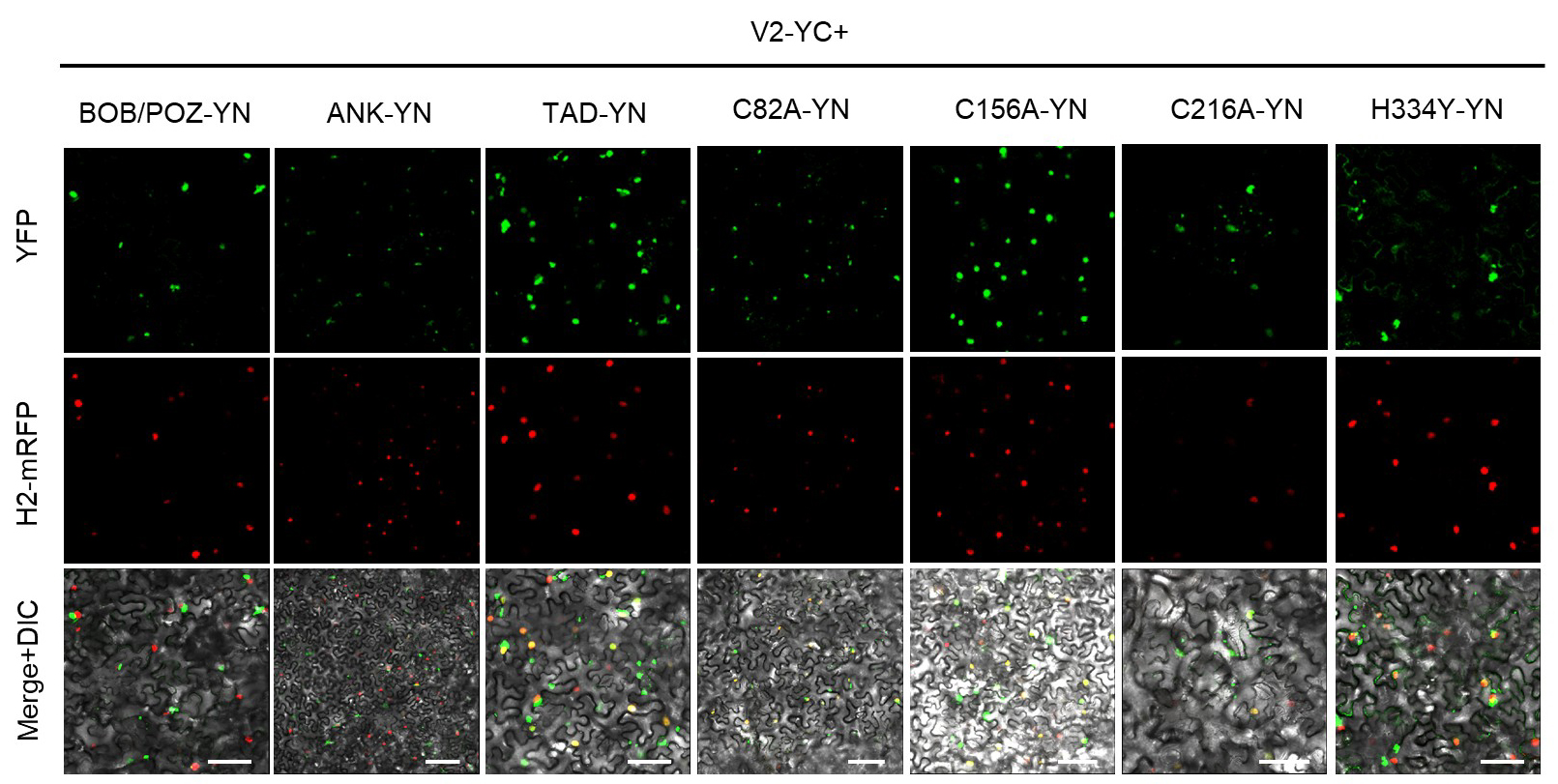
**

**Figure S8.** **BiFC assay for the interactions between V2-YC and YN-tagged truncated or point mutants of NPR1 in *N. benthamiana* epidermal cells.**

Proteins were expressed by agroinfiltration and photographs were taken at 2 dpi. The nuclei are indicated by mRFP-tagged histone H2 (H2-mRFP). Scale bars = 50 μm.


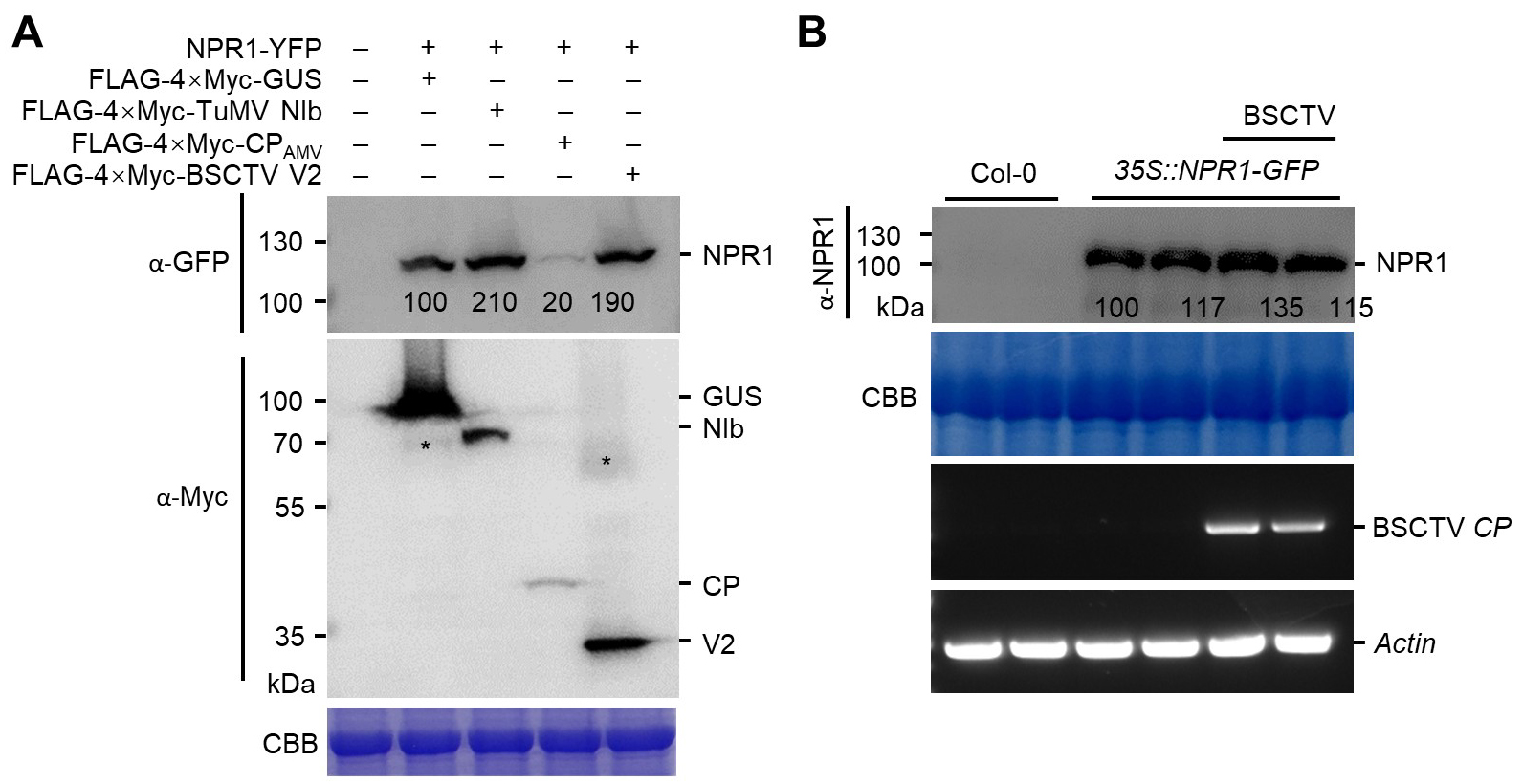


**Figure S9. V2 does not affects NPR1 stability.**

(A) Western blotting showing the accumulation of NPR1-YFP in *N. benthamiana* epidermal cells coexpressing FLAG-4×Myc-tagged GUS, TuMV NIb, AMV CP, or BSCTV V2 at 2 dpi with α-GFP and α-Myc. (B) Immunoblots for the accumulation of NPR1 in mock or BSCTV-infected *35S::NPR1-GFP* seedlings.


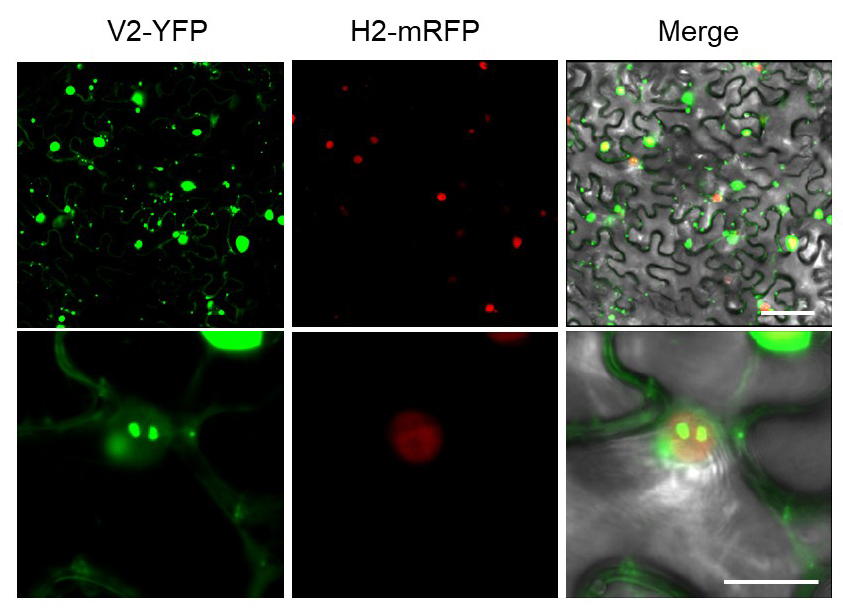


**Figure S10.** **Confocal microscopic photos of *N. benthamiana* epidermal cells expressing V2-YFP.**

The nuclei are indicated by mRFP-tagged histone H2 (H2-mRFP). The lower panels showing an enlarged nucleus. Scale bars = 50 μm (top panel) or 20 μm (bottom panel).

**
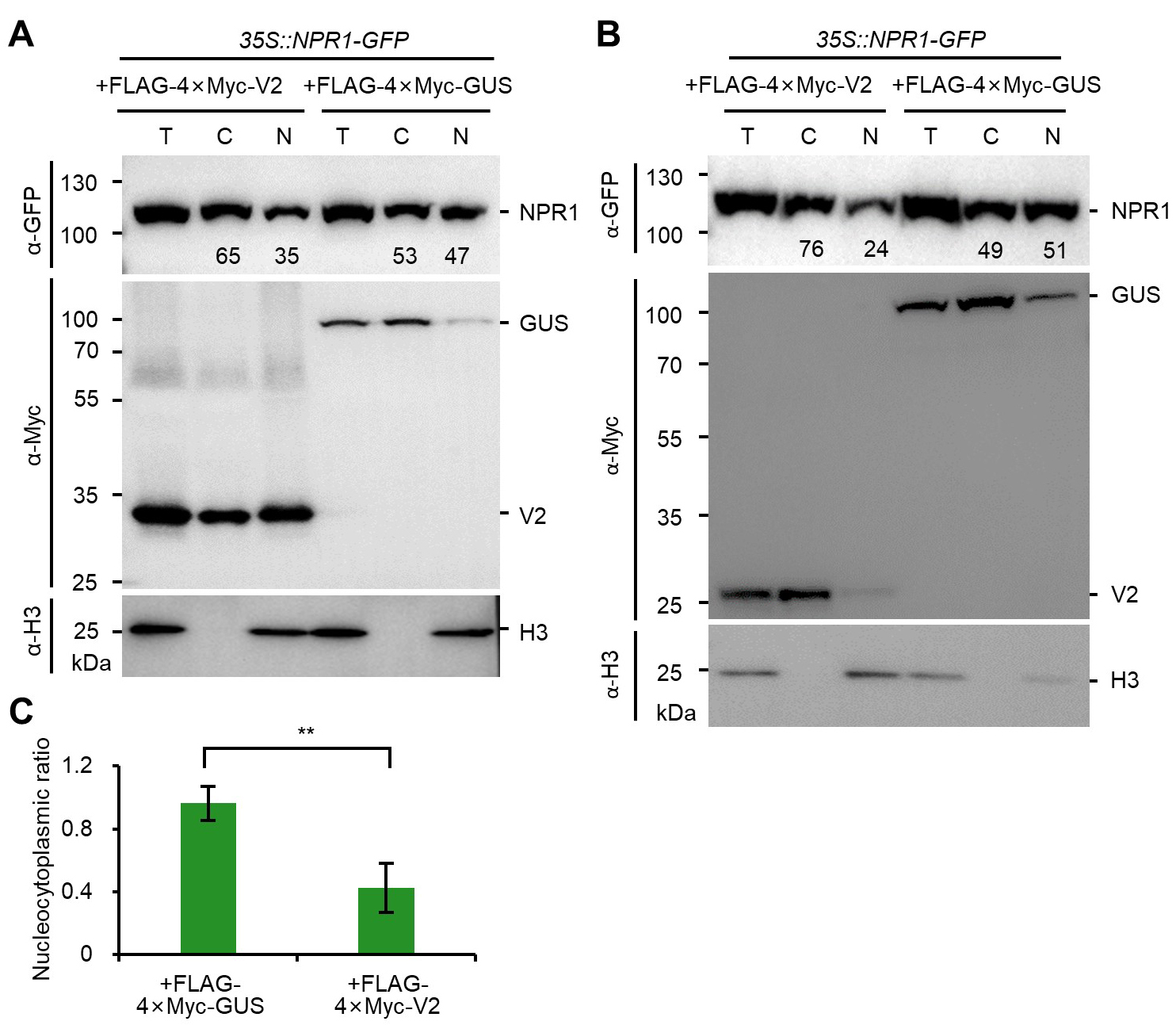
**

**Figure S11.** **BSCTV V2 affects** **NPR1 nucleocytoplasmic distribution in Arabidopsis.**

Western blot showing the accumulation of NPR1-GFP in the supernatant (cytoplasm) and pellets (nucleus) of cell lysates from *35S::NPR1-GFP* agroinfiltrated with FLAG-4×Myc-V2 or FLAG-4×Myc-GUS for 2 days. Histone H3 (H3) was detected using anti-H3 antibody. The number under each lane indicates the relative intensity of the band to the control. Panels A and B represent two independent repeats. T, total protein; C, cytoplasm; N, nucleus.

**
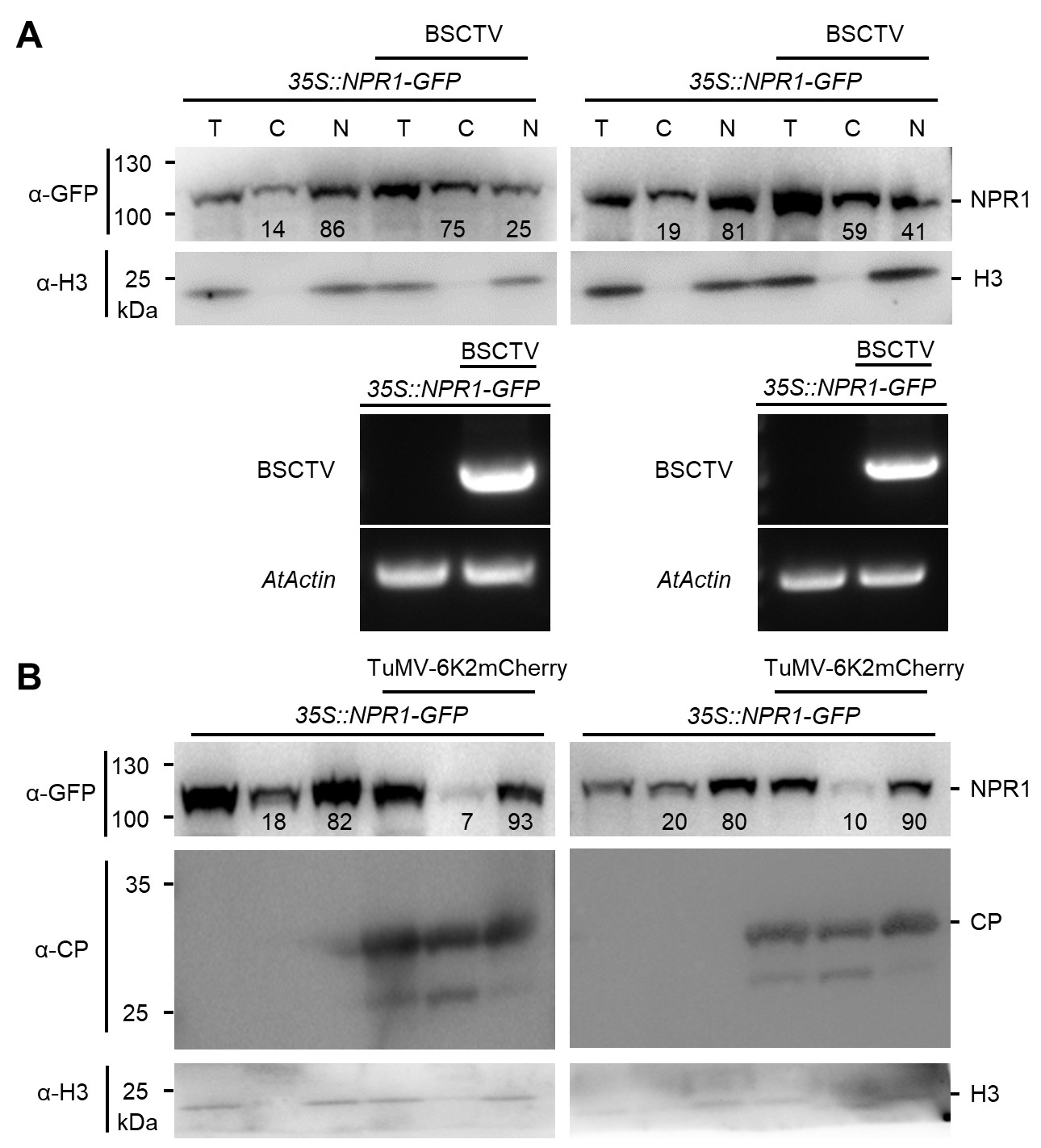
**

**Figure S12.** **ALY increases the effects of V2 on NPR1 nucleocytoplasmic distribution.**

Western blotting showing the accumulation of NPR1-YFP in the supernatant (cytosol) and pellets (nucleus) of centrifugally separated cell lysates of *N. benthamiana* leaves coexpressing NPR1-YFP, FLAG-4×Myc-V2 plus FLAG-4×Myc-NbALY or FLAG-4×Myc-GUS. Histone H3 (H3) was detected with anti-H3 antibodies. Number under each lane indicate the relative intensity of the band to the control. Panels A and B represent two independent repeats. T, total protein; C, cytosol; N, nucleus.

**
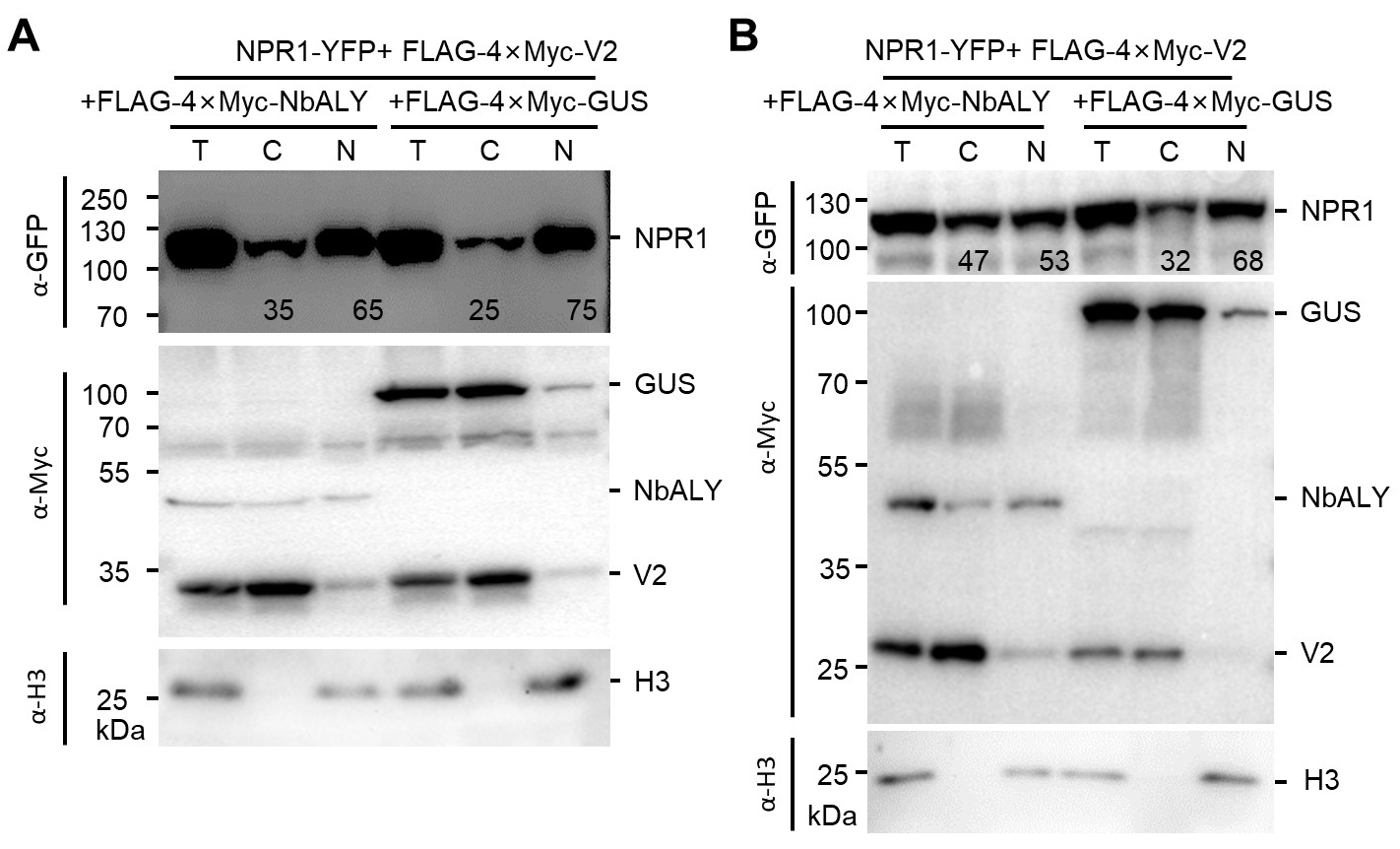
**

**Figure S13.** **ALY increases the effects of V2 on NPR1 nucleocytoplasmic distribution in Arabidopsis.**

Western blot showing the accumulation of NPR1-GFP in the supernatant (cytoplasm) and pellets (nucleus) of cell lysates from *35S::NPR1-GFP* agroinfiltrated with FLAG-4×Myc-V2 plus FLAG-4×Myc-NbALY or FLAG-4×Myc-GUS for 2 days. Histone H3 (H3) was detected using anti-H3 antibody. The number under each lane indicates the relative intensity of the band to the control. Panels A and B represent two independent repeats. T, total protein; C, cytoplasm; N, nucleus.

**
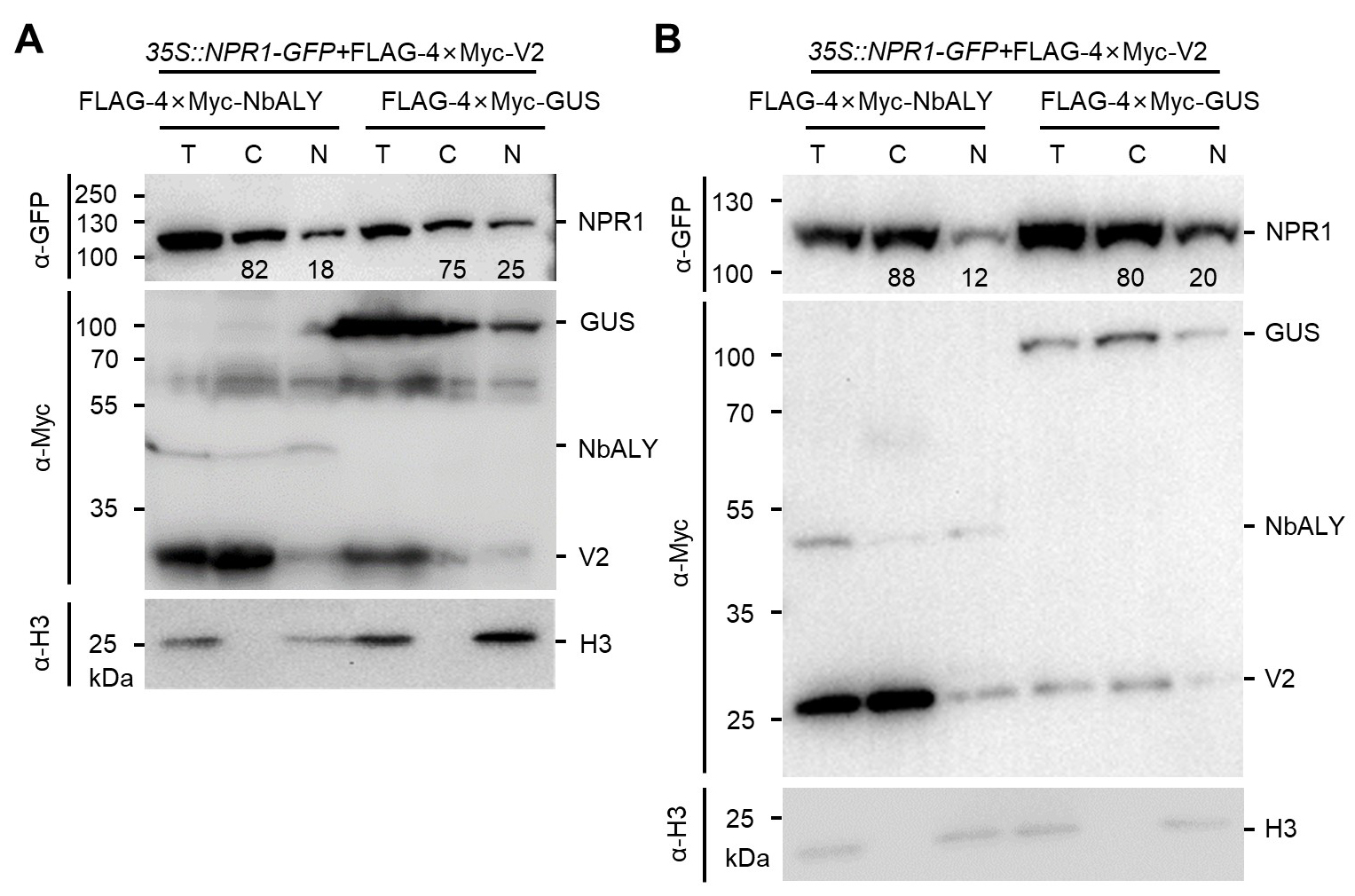
**

**Figure S14.** **Western blotting showing the nucleocytoplasmic distribution of NPR1 in *35S::NPR1-GFP* at the presence of BSCTV and TuMV-GFP.**

(A) Western blot showing the accumulation of NPR1-GFP in the supernatant (cytoplasm) and pellets (nucleus) of cell lysates from *35S::NPR1-GFP* infected by BSCTV at 10 dpi. Histone H3 (H3) was detected using anti-H3 antibody. BSCTV was confirmed by PCR and *AtActin II* was used as an DNA loading control (Bottom panel). The number under each lane indicates the relative intensity of the band to the control. The left and right panels represent two independent repeats. (B) Western blot showing the accumulation of NPR1-GFP in the supernatant (cytoplasm) and pellets (nucleus) of cell lysates from *35S::NPR1-GFP* infected by TuMV-6K2mCherry at 10 dpi. Histone H3 (H3) was detected using anti-H3 antibody. TuMV-6K2mCherry was confirmed by Western blotting using polyclonal antibodies against TuMV CP (α-CP). The number under each lane indicates the relative intensity of the band to the control. The left and right panels represent two independent repeats.

**
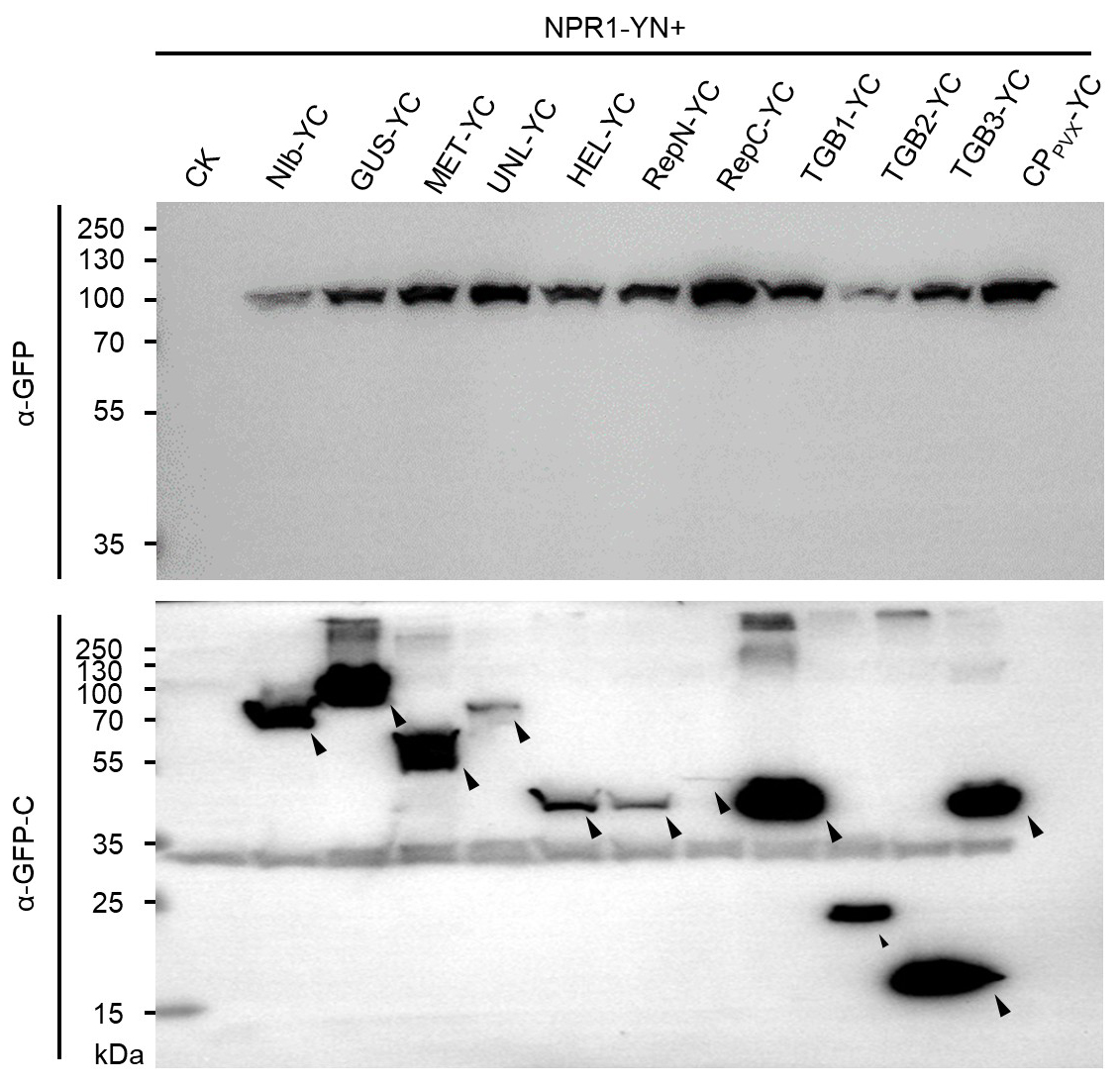
**

**Figure S15.** **Immunoblots for the expression of recombinant proteins Fig. 7A.**

NPR1-YN was detected by α-GFP and YC-tagged recombinant proteins were detected by α-GFP-C. The positions of NIb-YC, GUS-YC, MET-YC, UNL-YC, HEL-YC, RepN-YC, RepC-YC, TGB1-YC, TGB2-YC, TGB3-YC, and CP-YC are indicated by black arrow-heads, respectively.

**
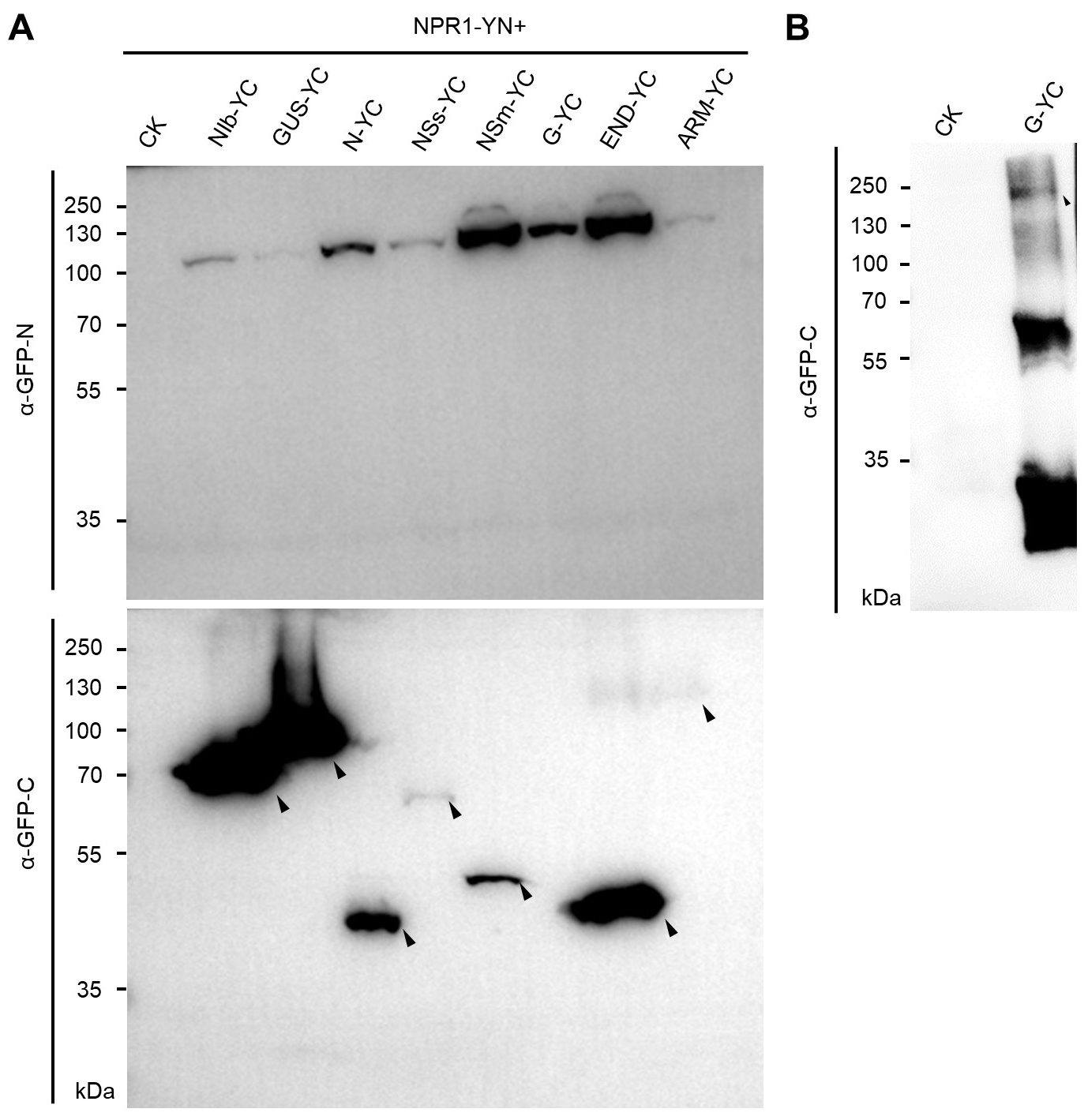
**

**Figure S16. Immunoblots for the expression of recombinant proteins Fig. 7E.**

(A) NPR1-YN was detected by α-GFP and YC-tagged recombinant proteins were detected by α-GFP-C. (B) Immunoprecipitation and Western blotting for the expression of 1a-YC. Due to low expression level, G-YC was affinity purified by anti-FLAG M2 affinity gel and then analyzed by α-GFP-C. The position of recombinant proteins are indicated by black arrow-heads.
